# Spatiotemporal analysis of microbial community dynamics during seasonal stratification events in a freshwater lake (Grand Lake, OK, USA)

**DOI:** 10.1371/journal.pone.0177488

**Published:** 2017-05-11

**Authors:** Jessica M. Morrison, Kristina D. Baker, Richard M. Zamor, Steve Nikolai, Mostafa S. Elshahed, Noha H. Youssef

**Affiliations:** 1 Department of Microbiology and Molecular Genetics, Oklahoma State University, Stillwater, OK, United States of America; 2 Grand River Dam Authority (GRDA), Vinita, OK, United States of America; Cairo University, EGYPT

## Abstract

Many freshwater lakes undergo seasonal stratification, where the formation of phototrophic blooms in the epilimnion and subsequent sedimentation induces hypoxia/anoxia in the thermocline and hypolimnion. This autochthonously produced biomass represents a major seasonal organic input that impacts the entire ecosystem. While the limnological aspects of this process are fairly well documented, relatively little is known regarding the microbial community response to such events, especially in the deeper anoxic layers of the water column. Here, we conducted a spatiotemporal survey of the particle-associated and free-living microbial communities in a warm monomictic freshwater reservoir (Grand Lake O’ the Cherokees) in northeastern Oklahoma, USA. Pre-stratification samples (March) harbored a homogeneous community throughout the oxygenated water column dominated by typical oligotrophic aquatic lineages (acl clade within Actinobacteria, and *Flavobacterium* within the Bacteroidetes). The onset of phototrophic blooming in June induced the progression of this baseline community into two distinct trajectories. Within the oxic epilimnion, samples were characterized by the propagation of phototrophic (*Prochlorococcus*), and heterotrophic (Planctomycetes, Verrucomicrobia, and Beta-Proteobacteria) lineages. Within the oxygen-deficient thermocline and hypolimnion, the sedimentation of surface biomass induced the development of a highly diverse community, with the enrichment of Chloroflexi, “Latescibacteria”, Armatimonadetes, and Delta-Proteobacteria in the particle-associated fraction, and Gemmatimonadetes and “Omnitrophica” in the free-living fraction. Our work documents the development of multiple spatially and temporally distinct niches during lake stratification, and supports the enrichment of multiple yet-uncultured and poorly characterized lineages in the lake’s deeper oxygen-deficient layers, an ecologically relevant microbial niche that is often overlooked in lakes diversity surveys.

## 1. Introduction

Freshwater lakes are biologically complex ecosystems that contribute several economic and societal services and provide habitats for a wide range of micro- and macro-organisms [1]. Microbial community characterization in freshwater lakes has been the subject of a wide range of studies using culturing approaches [2–17], culture-independent diversity surveys [2, 7, 8, 13, 14, 18–29], and–omics based investigations [8, 30–43]. These efforts have generated extensive inventories of microbial taxa inhabiting such ecosystems. Further, the integration of sequence data with geochemical, climatic, and limnological data has provided valuable insights into factors controlling microbial community structure in lakes, e.g. patterns of organic carbon deposition, predator-prey interactions [44, 45], the overall geography of the lake [46], the trophic status of the lake [47], along with other abiotic factors [48, 49].

Organic carbon deposited in lake ecosystems could either be allochthonous (i.e., originating from an exogenous source, e.g. hydrocarbon contamination, agricultural, municipal, and industrial waste runoff), or autochthonous (i.e., originating from within the lake, e.g. due to carbon fixation by photosynthetic primary producers within the lake’s microbial community). Autochthonous deposition is often associated with the development of algal blooms, of which frequency and intensity are expected to increase in the future due to global patterns of increased nitrogen and phosphorous deposition from agricultural runoff and the expected rise in mean atmospheric temperature associated with global climate change [50].

Although algal blooms are formed due to the massive propagation of phototrophic microorganisms, multiple microbial communities of heterotrophs are subsequently stimulated by the increased carbon deposition in the lake ecosystem. The nature of interaction between these two metabolic groups of organisms during blooming events is complex and dynamic, and could range from mutualism, to commensalism, and even to parasitism [51]. Regardless of the nature of interaction, the microbial community stimulated by algal blooms could either be associated with the phycosphere, i.e. the microenvironment surrounding algal cells collectively made up of algal extracellular products that stimulate microbial growth [51–56], or could be free living and thrive on secreted organic matter or soluble metabolic low molecular weight products of the phycosphere [57].

The eventual fate of algal blooms plays an extremely important role in shaping the lake water chemistry and trophic status. Although a portion of the deposited organic carbon is processed by the surface heterotrophic microbial communities [58], a significant fraction of suspended particles of algal cells and the associated phycosphere community sinks to deeper layers in freshwater lakes. Indeed, it is estimated that algal cells represent up to 90% of sinking organic matters in stratified lakes [59–64].

Surprisingly, while multiple studies of the microbial community associated with algal blooms have been undertaken, the majority of this work has been conducted in marine ecosystems [57, 65–69]. More importantly, the majority of this work has focused on phycosphere development in epilimnion communities, with little to no effort conducted on the effect of blooming and organic carbon deposition into freshwater lakes deeper layers. Such process is especially important in meromictic, and holomictic lakes, where the lack of upwelling results in greater accumulation of organic matter into the lake’s deeper anoxic layers. To our knowledge, few studies have provided detailed spatiotemporal analysis of microbial community dynamics in stratified lakes, and how the lake’s microbial communities respond to seasonal blooming events and organic carbon deposition from the oxic/photic to the anoxic/aphotic layers.

In this study, we present a detailed analysis of the microbial community of Grand Lake O’ the Cherokee (Grand Lake), a large reservoir in Northeastern Oklahoma. Grand Lake is a seasonally-stratified warm monomictic lake. While the water column freely mixes throughout the winter, both seasonal stratification and algal blooms occur in mid-spring through mid-autumn and result in increased carbon deposition and the development of hypoxia/anoxia in the lake thermocline and hypolimnion. We hypothesize that the microbial communities would be similar throughout the lake during mixing, and that differences are expected in the microbial community membership and composition as the lake stratifies, e.g. differences between epilimnion and thermocline communities and between thermocline and hypolimnion communities. Differences are also expected between free-living and particle-associated fractions. We further hypothesize that the magnitude of these differences would increase with stratification time, e.g. summer versus autumn. Our results highlight the highly diverse and dynamic nature of microbial communities that develop at various depths and seasons in response to geochemical and climatic variability, and identify lineages responsible for organic carbon turnover at various depths either as part of the direct phycosphere or the wider free-living microbial community.

## 2. Materials and methods

### 2.1 Site description and sampling

**Fig 1:**
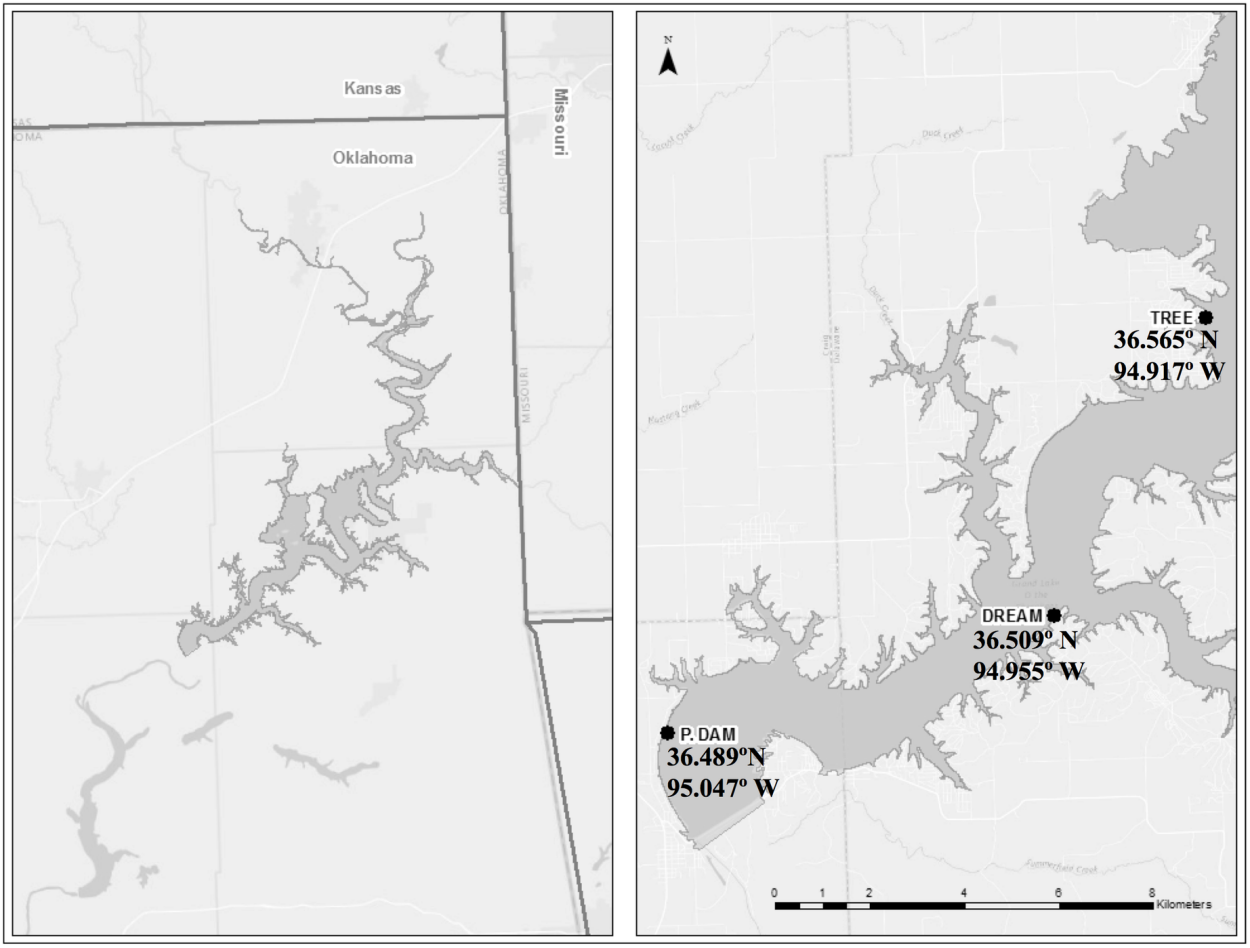
Geographical location of Grand Lake (right panel). The sites sampled for this study are shown in the left panel and their coordinates are noted. All three sites lie in the lacustrine area of the lake.

Grand Lake is a large (surface area: ~183 km^2^, volume: ~2.07 km^3^; at lake elevation 227 m (Pensacola Datum)) warm monomictic lake in Northeastern Oklahoma formed by the impoundment of the Grand River by the Pensacola Dam. The lake’s water quality has been continuously monitored by the Grand River Dam Authority (GRDA) since 2012. Grand Lake exhibits longitudinal zonation with riverine, transition, and lacustrine zones with a mean depth of 11 m and a maximum depth of 41 m near the dam [70]. The lake is characterized as eutrophic based on Carlson’s Trophic State Index [71]. Three sites in the southwestern reaches of the lake (in the lacustrine zone) were chosen for in-depth community characterization: P. Dam (36.489°N 995.047°W), Dream (36.509°N 94.955°W), and Tree (36.565°N 94.917°W) (Fig 1). The choice of the sites was based on prior observations that they undergo seasonal algal blooms and develop stratification with complete anoxia in the hypolimnion.

Water samples were obtained at the three above-mentioned sites in March 2015 (early spring prior to algal blooms, no stratification, completely oxygenated water column), June 2015 (early summer, during algal bloom, stratified water column), and September 2015 (late summer, post algal bloom, stratified water column with an increased particulate organic matter sedimentation to the thermocline and hypolimnion). Samples were collected from the epilimnion (1 m depth), thermocline (depth in June and September (S1 Table) was determined in the field based on temperature and dissolved oxygen concentration patterns), and hypolimnion (1 m off of the bottom) layers. Vertical profiles measuring environmental parameters (temperature, conductivity, salinity, depth, pH, oxidation-reduction potential, turbidity, Chlorophyll-a, dissolved oxygen, cyanobacteria (phycocyanin)) were taken using an YSI 6 Series Multiparameter sonde at each site during each sampling trip.

Sampling at depth was conducted using a 4-L Van Dorn bottle. One liter of lake water was obtained per sample. Water from depth was transferred to 1-L Nalgene bottles and kept on ice until transported to an onsite laboratory (sampling trips usually take between 6–8 hours depending on lake conditions). Upon arrival, the samples (1L each) were immediately processed for assessing the microbial community as well as other analytes. We used successive filtration to separate the particle-associated (PA) from the free-living planktonic (FL) communities. The particle-associated community was retained on 3-μm polycarbonate membrane filters (Millipore®), while the free-living community was obtained from the flow-through by an immediate second filtration through 0.2-μm polycarbonate membrane filters (Millipore®).

### 2.2 DNA extraction, PCR amplification, and Illumina sequencing

DNA was extracted from the 3-μm and 0.2-μm filters using GeneRite® DNA-EZ extraction kit (New Brunswick, NJ, USA) according to the manufacturer’s instructions. A total of 54 extractions (3 sampling events x 3 sites x 3 depths x 2 fractions (PA and FL)) were conducted. DNA obtained was quantified using Qubit® fluorometer (Life technologies®, Carlsbad, CA), and used as template for amplifying the V4 hypervariable region of 16S rRNA using the prokaryotic-specific primer pair 515F and 806R [72]. Products were sequenced using paired-end Illumina Miseq platform, as previously described [73]. Both PCR amplification and Illumina sequencing were conducted using the services of the Genomic Sequencing and Analysis Facility (GSAF) at the University of Texas at Austin. Out of the 54 samples, four yielded poor sequencing results (P. Dam_March_thermocline_PA, P. Dam_March_hypolimnion_FL, Tree_March_thermocline_PA, Tree_March_thermocline_FL) while one yielded a very small number of sequences (less than 200 sequences; P. Dam_June_surface_PA). Analysis was therefore conducted on 49 datasets as described below. The sequences are deposited in the SRA database under accession number SRP096639.

### 2.3 Sequence analysis

#### 2.3.1 Sequence processing, alignment, and taxonomy

We used mothur [74] for all sequence processing and analysis. Most of the analyses were conducted on the Cowboy server, a high-performance supercomputer housed at the Oklahoma State High Performance Computing Center (http://hpcc.it.okstate.edu). Most of the steps were derived from the MiSeq SOP available from the mothur website (http://www.mothur.org/wiki/MiSeq_SOP)). Briefly, raw sequences were screened to eliminate sequences with an average quality score < 25, sequences containing ambiguous bases, sequences with a homopolymer stretch greater than 8 bases, and sequences longer than 293 bp. All sequences were grouped in one file for a comprehensive analysis. Sequences were aligned using as a template the recreated Silva SEED alignment database downloaded from the mothur website. Aligned sequences were then filtered to remove columns that corresponded to ‘.’ or ‘-’ in all sequences. Filtered alignments were then subjected to a pre-clustering de-noising step (implemented in mothur) using a pseudo-single linkage algorithm with the goal of removing sequences that are likely due to sequencing errors [75]. Possible chimeric sequences were identified and removed using the command chimera.slayer [76] implemented through mothur. The aligned, filtered, de-noised, and chimera-free sequences were clustered into operational taxonomic units (OTUs) at 3% sequence divergence cutoff (the putative species level) as well as 10% sequence divergence cutoff (the putative order level) using the vsearch clustering method [77] employed through mothur. A shared file was created and was used for subsequent analyses. Sequence taxonomy was identified according to the Silva taxonomic outline (Release 123, https://www.arb-silva.de/).

#### 2.3.2 Diversity analyses

Rarefaction curve analysis and various metrics of species richness (number of observed OTUs at the putative species (OTUs_0.03_), as well as putative order (OTUs_0.1_) levels, Ace richness index), and diversity (Shannon diversity index) were performed on individual samples using the rarefaction.single and the summary.single commands in mothur. Since some of the alpha-diversity indices are dependent on the dataset sample size (e.g. number of observed OTUs_0.03_, number of observed OTUs_0.1_, Ace richness index, and Shannon diversity index), we used the sub.sample command in mothur to randomly select a number of sequences from each high-quality-sequence dataset equivalent to the number of sequences in the smallest dataset (n = 1376) and used the randomly selected sub-samples for the comparative alpha diversity analysis. Beta diversity indices (Bray-Curtis dissimilarity coefficient) were also calculated in mothur using the shared file created as explained above. Using Bray-Curtis dissimilarity indices, non-metric multidimensional scaling plots (NMDS plots) were constructed using the nmds command in mothur for visualization.

### 2.4 Statistical analysis

#### 2.4.1 Significance of physical and chemical characteristics change as a result of lake stratification

The significance of the changes in physical and chemical characteristics of the lake as a result of stratification was evaluated by comparing Student t-test (in cases where Shapiro-Wilks test for normality rejected the non-normal distribution for both datasets compared), or Wilcoxon ranked sum test (in cases where the hypothesis of normal distribution was rejected by the Shapiro-Wilks test for normality for either or both of the datasets compared) p-values.

#### 2.4.2 Statistical significance of the effect of lake stratification on microbial diversity and community structure

Rarefaction curve ranks (as a proxy for diversity) were correlated to the lake’s physical and chemical properties using Spearman correlation, and the significance of such correlations was tested by comparing the p-values. To study the significance of the effect of sampling season, sampling depth, the physical state of the sample (PA versus FL), and sampling site on the bacterial community structure, we performed an analysis of variance using both the multi response permutation procedure using the function mrpp, and permutational multivariate analysis of variance using the function Adonis in the R statistical package vegan [78] with the Bray-Curtis dissimilarity matrix as the input for community structure. Communities were further compared based on those factors that showed a significant effect on community structure in the analysis of variance tests. Using each one of these factors at a time, we tested the significance of difference in community structure (based on Bray-Curtis dissimilarity indices) by comparing Student t-test p-values corrected for multiple samples using Bonferroni correction as follows. For inter-time or inter-depth comparisons, since 6 possible pairwise comparisons exist, we used Bonferroni correction for significant p-value = 0.0083, i.e. differences with p-values ≤ 0.0083 are considered significant, while for inter-sample-physical-state comparisons, since 3 possible pairwise comparisons exist, we used Bonferroni correction for significant p-value = 0.017, i.e. differences with p-values ≤ 0.017 are considered significant.

## 3. Results

### 3.1 Physical and chemical parameters

**Fig 2:**
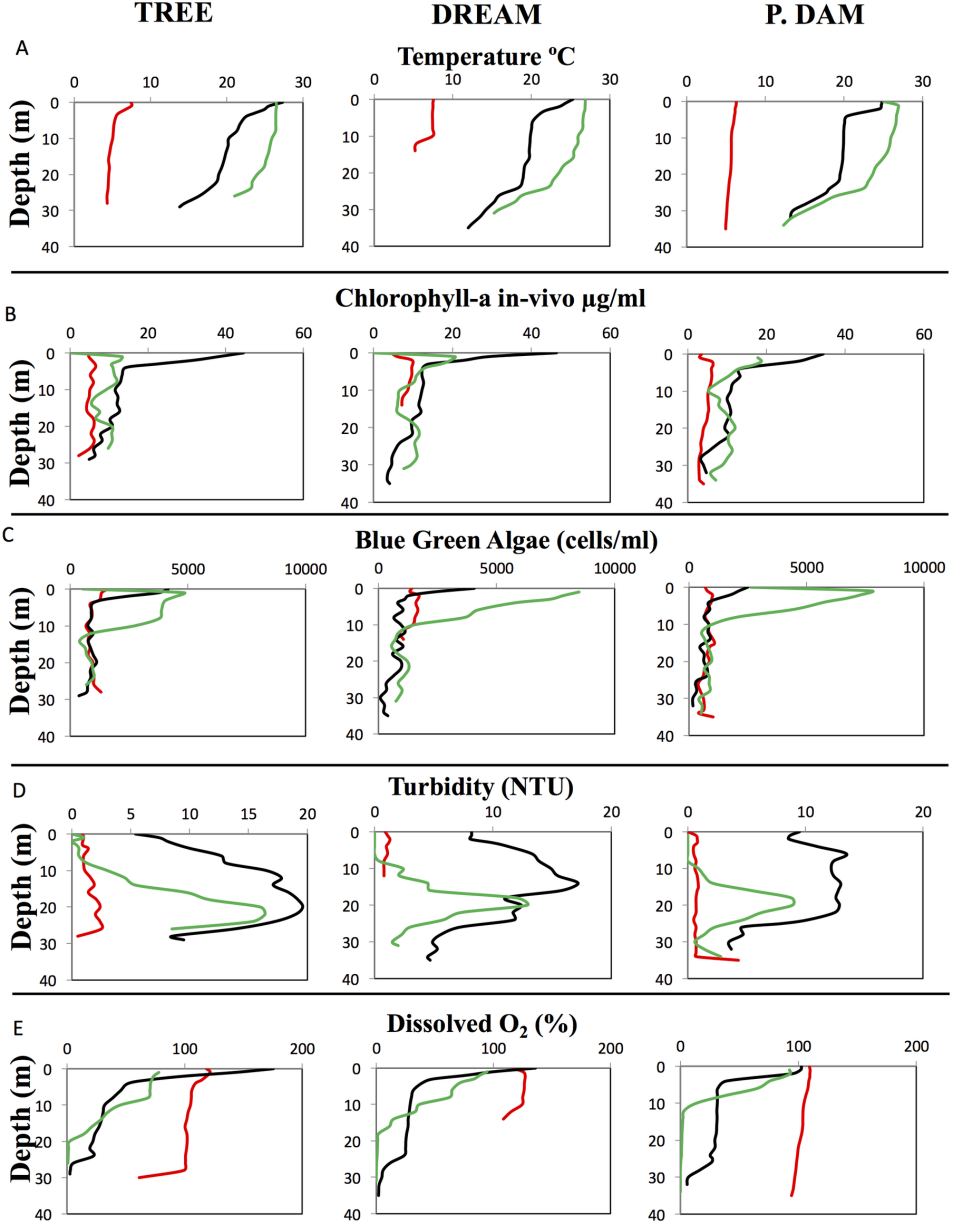
Grand Lake physical and chemical characteristics (X-axis) along depth (in meters) (Y-axis) and season (March, red; June, black; and September, green). Data are shown for the three sites sampled in this study (site name is shown on top).

A uniform physical pattern was observed across all three sites studied. Seasonal increase in water temperatures was observed from March to June to September (Fig 2A). This was associated with: 1. A marked increase in productivity in the epilimnion, as evident by a significant increase in surface chlorophyll-a levels (from 4.4±1.25–8.04±1.68 μg/L throughout the water column in March, to 28.1±6.76–36.78±12.6 μg/L in surface layers (0–3 m) in June (Fig 2B) (Wilcoxon ranked sum test coefficient (W) = 0, p-value = 8.8x10^-8^); as well as an increase in cyanobacteria numbers between March and June in surface (0–3 m) samples (Wilcoxon ranked sum test coefficient (W) = 39, p-value = 3.1x10^-6^) (Fig 2C), 2. A significant increase in total suspended solids throughout the water column, with turbidity values increasing from 0.7±0.1–1.5±0.64 NTU in March to 9.8±3.5–13.5±4.75 NTU in June (Wilcoxon ranked sum test coefficient (W) = 2, p-value = 2.2x10^-16^). In September, turbidity decreased back to pre-stratification levels in the epilimnion (approximately 8–10 meters deep) (Wilcoxon ranked sum test coefficient (W) = 891.5, p-value = 0.4242), but remained high at depths >10 meters (Wilcoxon ranked sum test coefficient (W) = 94.5, p-value = 6.97x10^-11^) suggesting the deposition of suspended solids into the deeper hypoxic and anoxic layers (Fig 2D). 3. The transition from an oxygenated, completely mixed, water column in March with dissolved oxygen concentrations ranging from 102.9±5.28%–122.4±6.18% throughout the water column (Fig 2E) into a stratified water column in June, where the dissolved O_2_ levels decreased with depth from highly oxic (90.1±37.2%–99.9±4.1%) in the epilimnion (top 6 meters) to moderately hypoxic (27.2±6.3%–29.4±4.8%) in the metalimnion (6–24 m deep) to highly hypoxic (3.48±1.3%–6.25±0.78%) in the hypolimnion (24–28 m). In September, the water column was highly stratified, with an oxic surface layer (1–8 m deep) (72.4±3.23%–82.5±12%), hypoxic metalimnion (10–18 m deep) (24.03±12.8%–28.8±12%), and anoxic hypolimnion (deeper than 18–20 m) (0.7±0.7%–1±1.05%) (Fig 2E).

### 3.2 Diversity patterns

**Fig 3:**
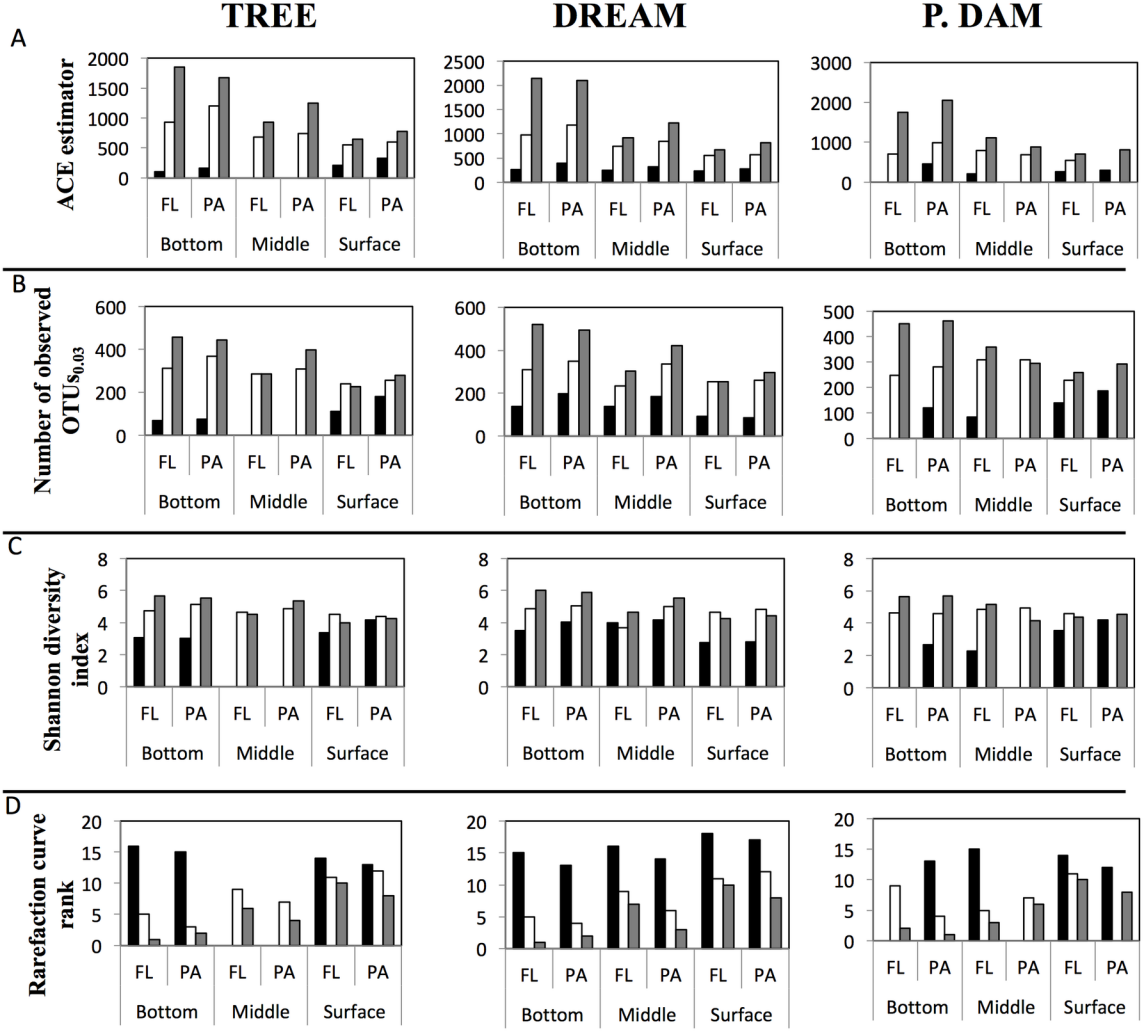
Diversity estimates calculated for each of the datasets obtained and shown for the three sites sampled in this study (site name is shown on top). (A) ACE species richness estimator, (B) the number of observed OTUs _0.03_, (C) Shannon diversity index (A-C all normalized to the number of sequences in the smallest dataset), and (D) rarefaction curve ranking (with 1 being the most diverse). Results are grouped first by sampling depth, then by the physical state of the sample (free-living (FL) versus particle-associated (PA)) and shown across the sampling season (March, Black bars; June, White bars; September, Grey bars).

Multiple diversity measures (OTUs_0.03_, ACE richness estimator, Shannon diversity index (all normalized to the sample size of the smallest dataset), and rarefaction curve-based diversity rankings) were used to compare diversity across datasets (Fig 3, S2 Table). In general, March samples were the least diverse, and diversity levels increased progressively during (June), and post (September) stratification regardless of the site, depth, and physical state of the sample (FL versus PA). Indeed, all physical and chemical progression patterns from March–June–September described above, e.g. lower dissolved oxygen concentration, higher temperature, and particulate matter deposition into lower layers were positively correlated to the level of diversity observed (Table 1). Finally, within the majority of June and September samples, the PA community was more diverse than the FL community (Fig 3, S2 Table).

**Table 1:** Spearman rank correlation coefficients (σ) of rarefaction diversity rank to various physical and chemical measures, and the p-values for the significance of such correlations. Significant correlations are shown in boldface.

	Dream			P. Dam		Tree	
	σ		P-value	σ	P-value	σ	P-value
Temperature (°C)	**-0.49**	**0.04**		-0.42	0.1	-0.37	0.1
Dissolved Oxygen (%)	**0.85**	**7.8 x10**^**-6**^		**0.87**	**2.5 x10**^**-5**^	**0.67**	**0.004**
Turbidity (NTU)	**-0.47**	**0.04**		-0.48	0.07	**-0.63**	**0.009**
Chl-a in-vivo (μg/L)	0.02	0.92		**-0.54**	**0.04**	-0.28	0.28

### 3.3 Community structure patterns.

Analysis of variance using the mrpp function as well as the Adonis function in R both showed that sampling time (mrpp p-value = 0.001, Adonis p-value = 0.01), and depth (mrpp p-value = 0.001, Adonis p-values = 0.01) significantly affected community structure. The effect of sampling depth was dependent on the sampling time (p-value = 0.04). Adonis analysis showed that sampling depth and sampling time explained ~29% of variance. On the other hand, the physical state of the sample (mrpp p-value = 0.132, Adonis p-value = 0.07), and the sampling site (mrpp p-value = 0.987, Adonis p-value = 0.81) had a non-significant, albeit variable, effect on community structure. Accordingly, we sought to study the individual effects that sampling time or depth (both being the factors identified by the analysis of variance test as significantly affecting community structure) had on the community structure. Since the physical state of the sample (PA versus FL) showed a moderate p-value in the analysis of variance test, we also opted to study its individual effect on community structure.

**Fig 4:**
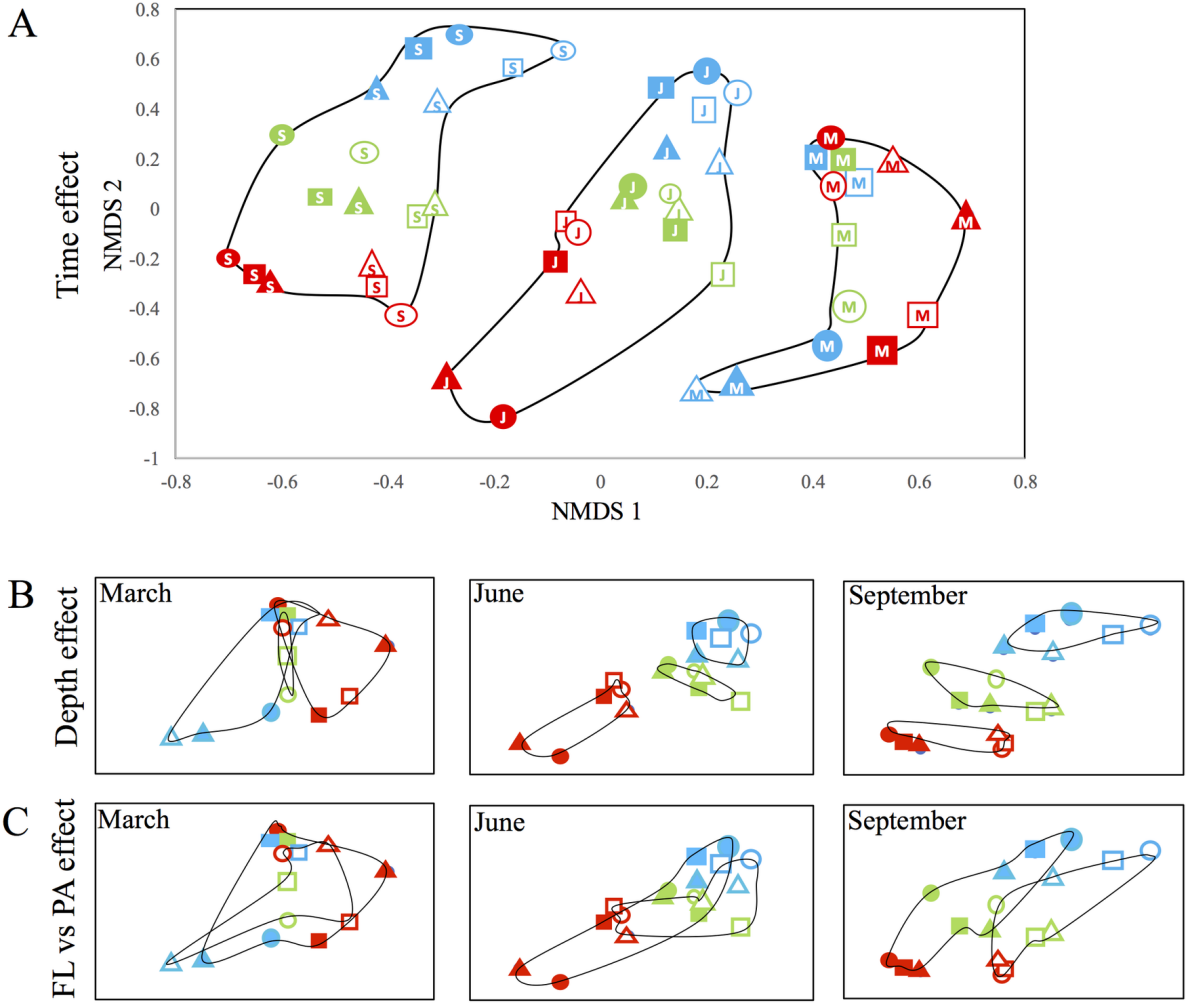
Microbial community structure analysis shown as non-metric multidimensional scaling (NMDS) plots based on Bray Curtis dissimilarity indices (at the species level (0.03)) for pairwise differences between datasets originating from the sites Tree (Circles), P. Dam (Squares), and Dream (Triangles). The sampling depth is denoted by color; epilimnion (red), thermocline (green), and hypolimnion (blue), the sample physical state is denoted by open (for free-living) and closed (for particle-associated) symbols, and the sampling time is shown by letters inside the symbols; March (M), June (J), and September (S). Sampling time (A) had the greatest effect on community structure where no overlap was observed between samples originating from different months. The same NMDS plot in (A) is shown in panels B and C but truncated to only show samples from a single sampling month (March, June, or September) as indicated in the top left corner of the Fig. To facilitate visualization of the depth (panel B) and the sample physical state (panel C) on community structure, black lines were added to surround all samples from the same depth (panel B) or with the same physical state (panel C). Samples were grouped by the sampling depth (B) in June (middle panel), and September (right panel) but not in March (left panel). Note the accentuation of the sampling depth effect on community structure as time increases from June to September. Finally, the sample physical state (C) affected the community structure in June (middle panel), and to a larger extent in September (right panel), where a clear separation of the PA (closed symbols) and FL (open symbols) communities is starting to form.

Community structures were compared across all different sampling times (March, June, and September), depths (epilimnion, thermocline, and hypolimnion), and physical states (FL versus PA) using Bray-Curtis dissimilarity indices. NMDS plots confirmed that the sampling site had no effect on the community structure (Fig 4A). Pairwise comparisons of the community structure of samples within the same sampling depth and sample physical state but from different months revealed that communities grouped by sampling time (Fig 4A). Average Bray Curtis dissimilarity indices are discussed below and shown in S3 Table. Community structure of June samples was distinct from the corresponding samples in March (average Bray Curtis dissimilarity index = 0.81±0.14). Similarly, community structure of September samples was distinct from corresponding samples in June (average Bray Curtis dissimilarity index = 0.75±0.13). The largest difference in community structure was between March samples and the corresponding September samples (average Bray Curtis dissimilarity index = 0.91±0.08). Indeed, Student t-test showed that the difference in community structure between March and September samples was more significant compared to March-June (p-value = 0.002) or June-September samples (p-value = 0.0001). In addition, within this broad sampling time-dependent clustering pattern, distinct depth-dependent, and physical-state-dependent (free-living (FL) vs particle-associated (PA)) community structure variabilities were observed post stratification, with the effect increasing with time. Pairwise comparisons of the community structure of samples within the same sampling time and sample physical state but from different depths revealed that post-stratification communities (June and September) grouped by depth (Fig 4B, S3 Table). In June, epilimnion samples showed an average Bray Curtis dissimilarity index of 0.77±0.18 to the thermocline samples, and an average Bray Curtis dissimilarity index of 0.81±0.13 to the hypolimnion samples, while thermocline samples showed an average Bray Curtis dissimilarity index of 0.56±0.16 to the hypolimnion samples. Student t-test showed that the difference in community structure between epilimnion and hypolimnion communities was more significant than the difference between the thermocline and hypolimnion communities (p-value = 0.006), while the thermocline and epilimnion communities and the hypolimnion and thermocline communities were equally different (p-value = 0.02). In September, the differences in community structures were more pronounced especially in epilimnion samples. Epilimnion samples showed an average Bray Curtis dissimilarity index of 0.8±0.07 to the thermocline samples, and an average Bray Curtis dissimilarity index of 0.88±0.13 to the hypolimnion samples, while thermocline samples showed an average Bray Curtis dissimilarity index of 0.57±0.14 to the hypolimnion samples. Student t-test showed that the difference in community structure between the epilimnion and hypolimnion samples was more significant than the difference between thermocline and hypolimnion communities (p-value = 0.0002) and that the difference in community structure between the epilimnion and thermocline samples was more significant than the difference between thermocline and hypolimnion communities (p-value = 0.002). Student t-test also showed that the epilimnion and thermocline and the epilimnion and hypolimnion communities were equally different (p-value = 0.018).

Looking at the effect of the sample physical state on microbial community structure, pairwise comparisons of FL vs PA samples from the same sampling event, i.e. same sampling time and same depth demonstrated a high level of similarity in March samples (average Bray Curtis index = 0.39±0.06, S3 Table). In June, the differences in microbial community between PA and FL were significantly higher (p-value = 0.0004) when comparing epilimnion samples (average Bray Curtis index = 0.88±0.18, S3 Table) to thermocline and hypolimnion samples (average Bray Curtis dissimilarity index = 0.38±0.09, S3 Table). In September, a significantly higher level of dissimilarity between PA and FL samples was observed in thermocline and hypolimnion samples (average Bray Curtis dissimilarity index = 0.46±0.02, S3 Table), when compared to the PA and FL microbial community differences in the same strata in March (p-value = 0.003), but were equally different when compared to the same strata in June (p-value = 0.04). On the other hand, the high dissimilarity between PA and FL epilimnion communities that was observed in June (average Bray Curtis indices = 0.88±0.18, S3 Table) was greatly diminished in September (average Bray Curtis indices = 0.58±0.05, Fig 4 and S3 Table).

Collectively, NMDS plots demonstrated that sampling time represented the most important determinant of microbial community structure followed by the depth and less importantly the physical state of the sample. The role played by sampling depth and sample physical state was more apparent with time (Fig 4B and 4C), where in September a clear separation of epilimnion samples from hypolimnion and thermocline samples, and of PA from FL samples was observed.

### 3.4 Phylogenetic diversity of microbial communities identified in Grand Lake

**Fig 5:**
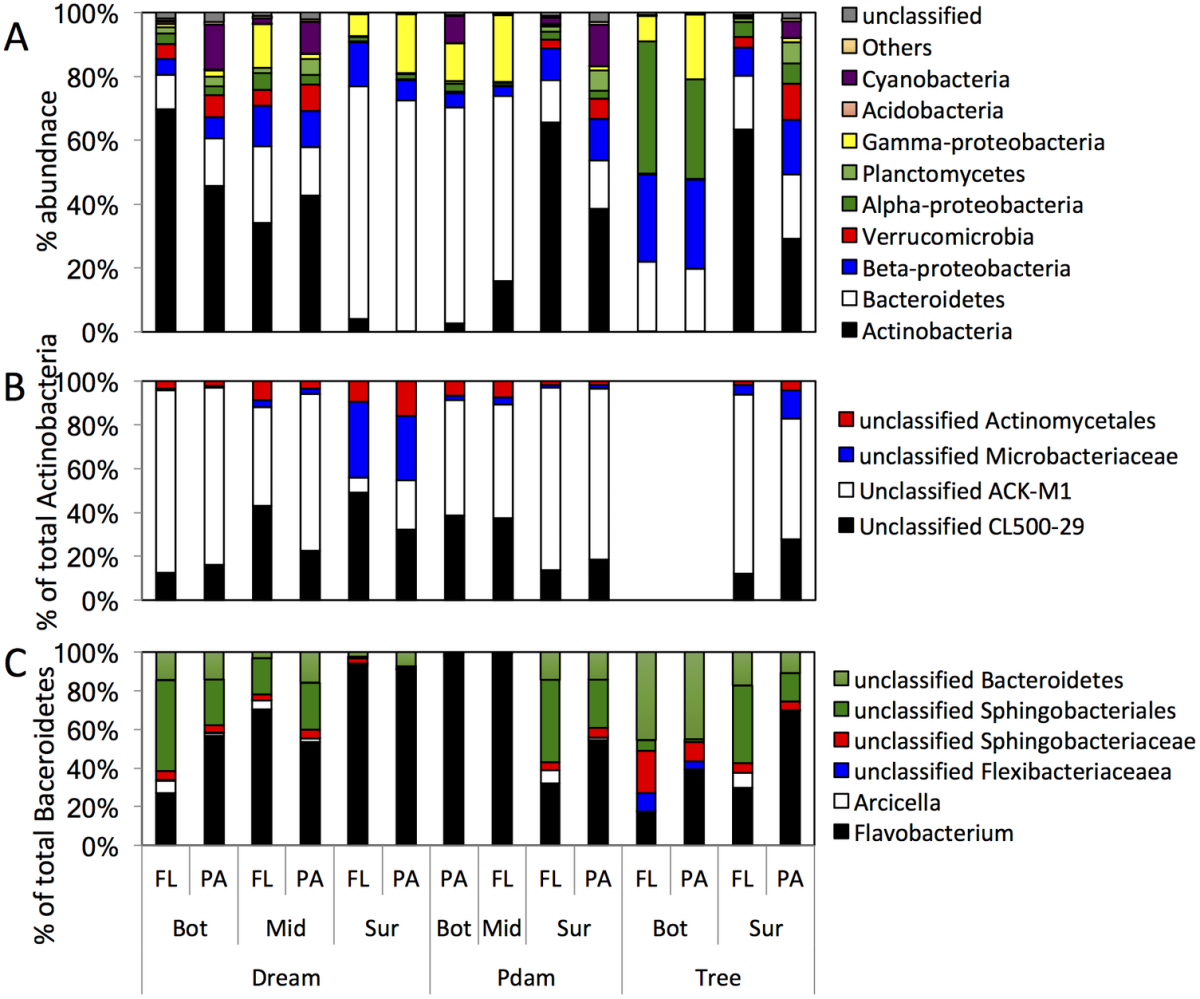
Microbial community composition in Grand Lake in March (pre-stratification) samples. Datasets are grouped on the X-axis first by the sampling site, then by the sampling depth (Bot, hypolimnion; Mid, thermocline; and Sur, epilimnion), then by the sample physical state (free-living, FL; particle-associated, PA). (A) The community composition is shown at the phylum level (or class level for Proteobacteria). “Others” denote all phyla with < 1% total abundance, and “unclassified” denote the sequences that could not be classified with accuracy at the phylum level. (B) Sub-class level classification of Actinobacteria. The Y-axis shows percentage within total Actinobacteria sequences identified. (C) Sub-class level classification of Bacteroidetes. The Y-axis shows percentage within total Bacteroidetes sequences identified.

**Fig 6:**
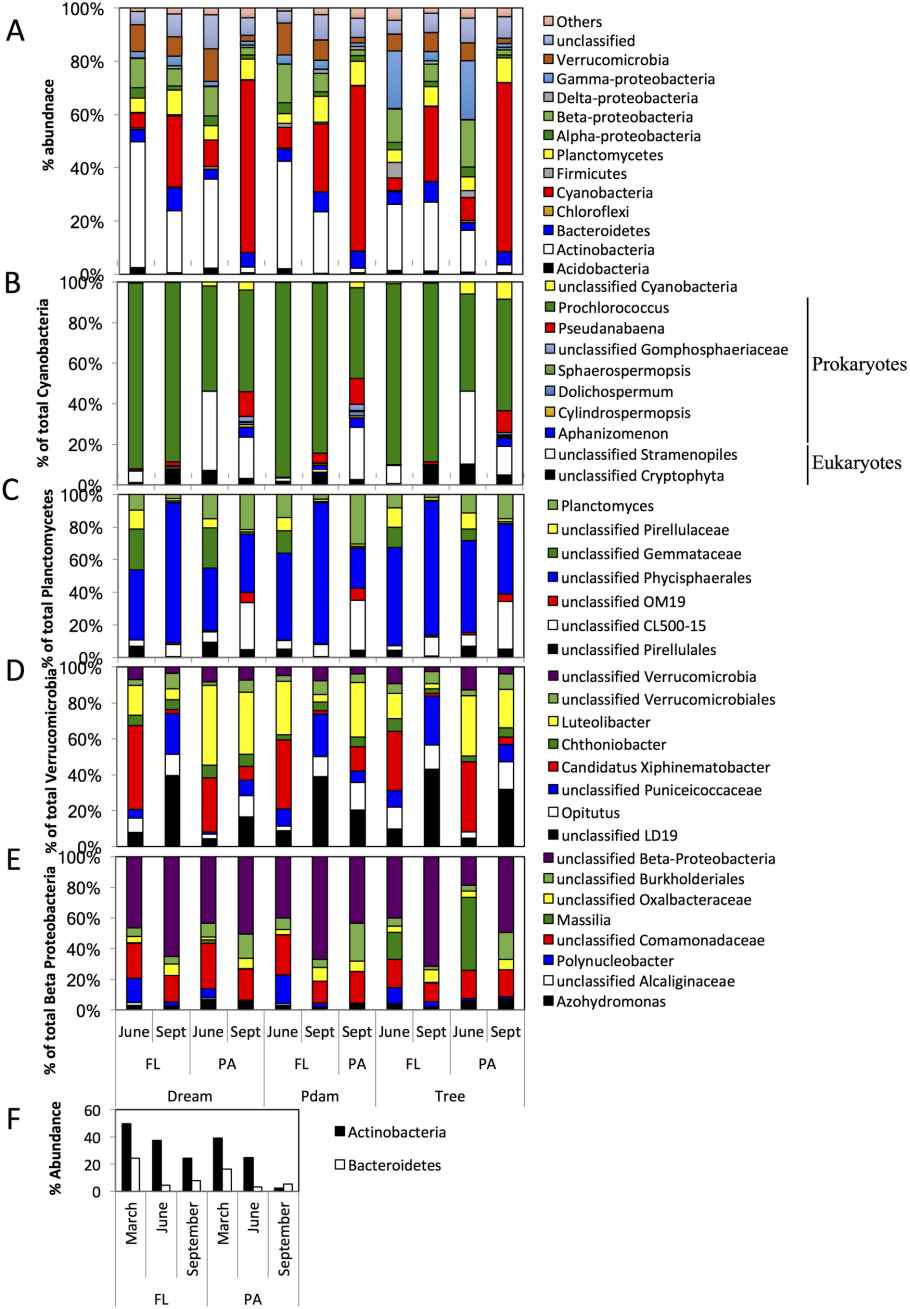
Epilimnion microbial community composition in Grand Lake in June and September when the lake was stratified. (A) The community composition is shown at the phylum level (or class level for Proteobacteria). “Others” denote all phyla with < 1% total abundance, and “unclassified” denote the percentage abundance of sequences that could not be classified with accuracy at the phylum level. (B-E) Sub-class level classification of (B) Cyanobacteria, (C) Planctomycetes, (D) Verrucomicrobia, and (E) Beta-Proteobacteria. The Y-axis in B-E shows percentage within total Phylum/Class sequences identified. The progressive decrease in Actinobacteria and Bacteroidetes average percentage abundance (across the three sites studied) is shown in (F) for the FL and the PA epilimnion communities. In (A-E) datasets are grouped on the X-axis first by the sampling site, then by the sample physical state (free-living, FL; particle-associated, PA), then by the sampling time (June; and September, Sept). Datasets in (F) are grouped on the X-axis first by the sample physical state (free-living, FL; particle-associated, PA), then by the sampling time (March, June, and September).

**Fig 7:**
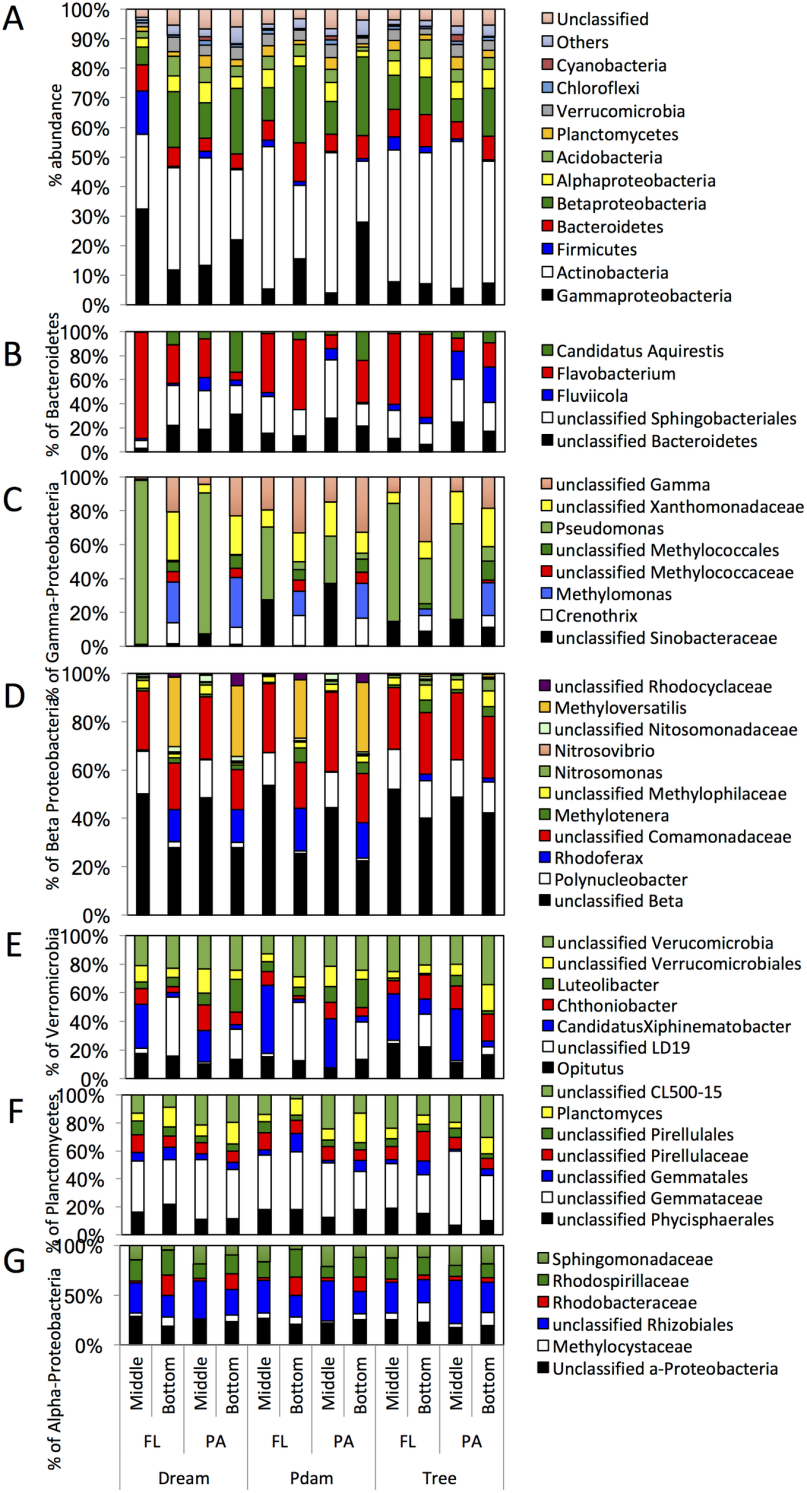
Thermocline and hypolimnion microbial community composition in June. The datasets are grouped on the X-axis first by the sampling site, then by the sample physical state (free-living, FL; particle-associated, PA), then by the sampling depth (Bottom, hypolimnion; Middle, thermocline). (A) The community composition is shown at the phylum level (or class level for Proteobacteria). “Others” denote all phyla with < 1% total abundance, and “unclassified” denote the percentage abundance of sequences that could not be classified with accuracy at the phylum level. (B-G) Sub-class level classification of (B) Bacteroidetes, (C) Gamma-Proteobacteria, (D) Beta-Proteobacteria, and (E) Verrucomicrobia, (F) Planctomycetes, and Alpha-Proteobacteria. The Y-axis in (B-G) shows percentage within total Phylum/Class sequences identified.

**Fig 8:**
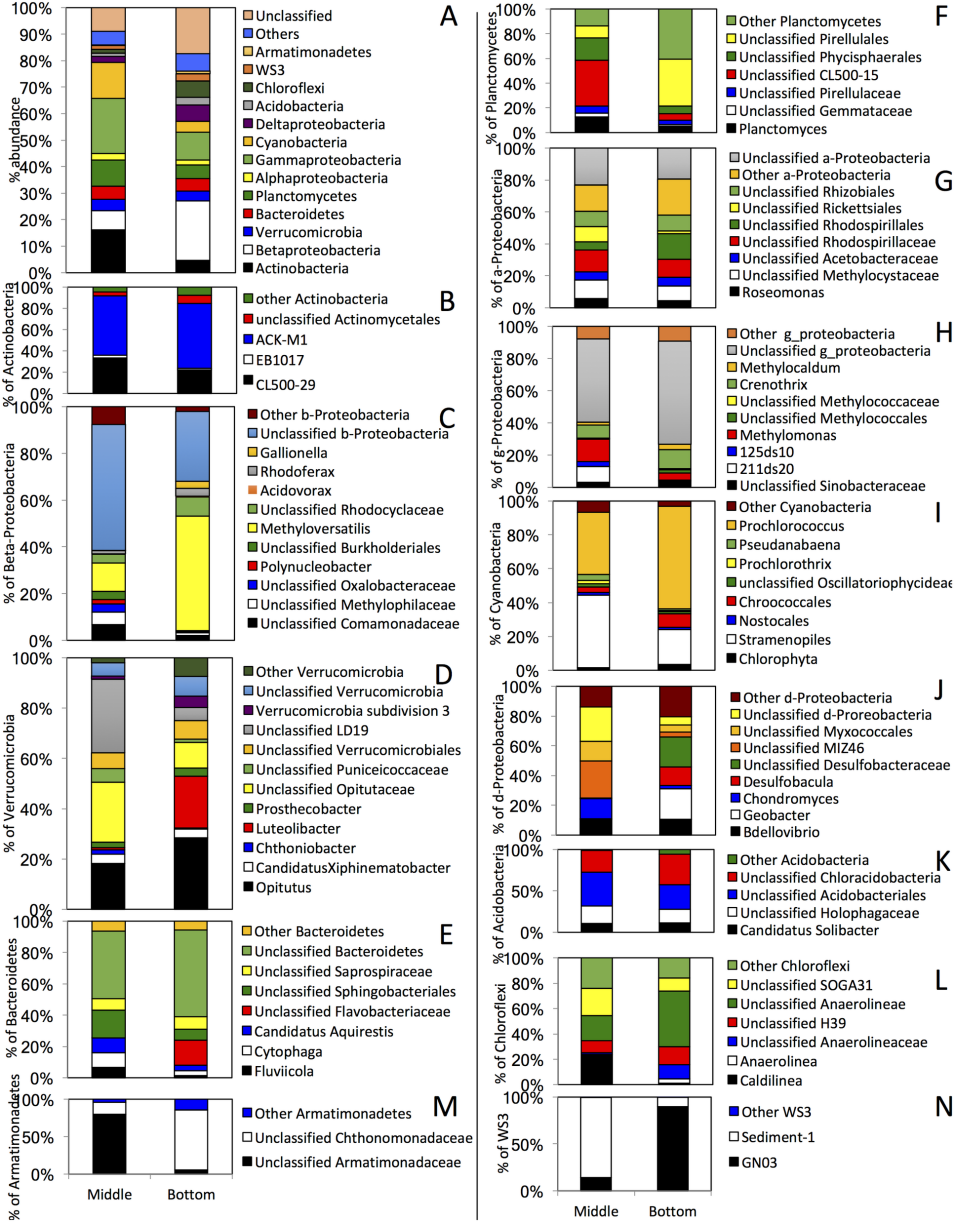
Thermocline (Middle) and hypolimnion (Bottom) particle-associated microbial community composition in September. Shown are the average percentage abundances across the three sites studied. (A) The community composition is shown at the phylum level (or class level for Proteobacteria). “Others” denote all phyla with < 1% total abundance, and “unclassified” denote the percentage abundance of sequences that could not be classified with accuracy at the phylum level. (B-N) Sub-class level classification of (B) Actinobacteria, (C) Beta-Proteobacteria, (D) Verrucomicrobia, (E) Bacteroidetes, (F) Planctomyetes, (G) Alpha-Proteobacteria, (H) Gamma-Proteobacteria, (I) Cyanobacteria, (J) Delta-Proteobacteria, (K) Acidobacteria, (L) Chloroflexi, (M) Armatimonadetes, and (N) WS3. The Y-axis in (B-N) shows percentage within total Phylum/Class sequences identified.

A total of 54 distinct bacterial phyla and candidate phyla (15 phyla ≥ 0.1% abundance), 13,800 OTU_0.03_ and 3,248 OTU_0.10_ were identified within all samples analyzed in this study. Based on the geochemical, diversity, and community structure patterns observed above (S1, S2 and S3 Tables, Figs 2–4), we recognize three different major groupings into which these samples can fit: 1. The homogeneous baseline microbial community identified in the pre-stratification samples in March (Fig 5), 2. The aerobic phycosphere epilimnion community developed in response to blooming where the onset of primary productivity elicited the formation of distinct PA and FL communities (Fig 6), and 3. The thermocline and hypolimnion microbial communities’ response to stratification, where the development of anoxia and deposition of carbon and particulates of organisms created a distinct community to degrade this input under the newly-formed anaerobic conditions (Figs 7 and 8). Below, we present a detailed analysis of the phylogenetic makeup of each of these communities.

#### 3.4.1. Microbial lake community prior to stratification/eutrophication (March)

All March samples were dominated by a few phyla, with members of the Actinobacteria and Bacteroidetes collectively representing the majority (49.26–80.62%) of sequences in March datasets (Fig 5A). While differences in relative proportion of Actinobacteria and Bacteroidetes were observed across sites, e.g. Actinobacteria dominated hypolimnion and thermocline samples of Dream (34.2–69.7%), and epilimnion samples in P. Dam and Tree (29.1–65.7%), while Bacteroidetes dominated hypolimnion and thermocline samples of P. Dam and Tree (19.5–67.6%), and epilimnion samples in Dream (~73%) (Fig 5A); the overall pattern of dominance of a few taxa within these two phyla was similar across all samples (Fig 5B and 5C). The majority of Actinobacteria sequences were members of the uncultured families CL500-29 (acl-A4) and the ACK-M1 (acl-A1) within the acl clade (Fig 5B), a universally distributed and ubiquitous lineage in freshwater lakes [21, 49]. While still an uncultured lineage, recent single cell genomics studies [39] predicted a heterotrophic carbohydrate-degrading potential for member of this lineage with the capacity to fix CO_2_ possibly in the absence of available organic carbon sources [39]. On the other hand, the majority of Bacteroidetes belonged to the genera *Flavobacterium* (representing 16.74-~99% of all Bacteroidetes members in March samples), *Arcicella* (0.04–7.1% of all Bacteroidetes, with abundances > 5% in 3 samples), as well as unclassified members of the Sphingobacteriales (0.67–42.1% of all Bacteroidetes, with abundances > 10% in 10 samples) (Fig 5C). All three Bacteroidetes lineages are typical freshwater inhabitants that thrive in oligotrophic as well as eutrophic lakes, possibly mediating high molecular weight dissolved organic matter degradation (e.g. *Flavobacterium* [79–81]), or especially adapted to oligotrophic conditions and low organic carbon concentration (e.g. *Arcicella* [82–84]).

In addition to Actinobacteria and Bacteroidetes, members of the Gamma-Proteobacteria, Planctomycetes, and Verrucomicrobia constituted a significant but less abundant fraction of the microbial community in all March samples (4.6–21.4% of total bacterial sequences) (Fig 5A). Finally, members of the Alpha- and Beta-Proteobacteria represented minor fraction of the March microbial community with one notable exception: These lineages constituted significant components of the microbial community in the hypolimnion layers of Tree site (27.4–27.9% of the total community for Beta-Proteobacteria, and 31.1–41.4% of the total community for Alpha-Proteobacteria (Fig 5A)). Remarkably, members of the Cyanobacteria, the main primary producers in many lakes (and also in Grand Lake as evident in subsequent sampling events), were only abundant (> 5%) in a few samples in March (n = 5) (Fig 5A), possibly due to the lower temperatures which are expected to limit the competitiveness of primary producers [85].

#### 3.4.2. Epilimnion community development in response to blooming

Phylogenetic analysis of the June and September epilimnion samples demonstrated the development of communities in both FL and PA fractions that are quite distinct from March samples (Fig 6A). The June/September epilimnion community structure had three distinct groups:

A. Primary producers (Fig 6B): This group includes both prokaryotic (Cyanobacteria) and eukaryotic (chloroplasts) phototrophs, and ranged in abundance from 4.66–9.94% of the total sequences in June, to 25.33–64.6% of the total sequences in September. The proportion of this group progressively increased from June to September in both PA (from 9.3±0.9% in June to 63.3±1.4% in September, Student t-test P-value = 6.9x10^-5^) and FL (from 5.97±1.6% in June to 26.7±1.4% in September, Student t-test P-value = 1.6x10^-5^) samples in all locations, and this increase was more pronounced in the PA fraction (Student t-test P-value = 5x10^-6^). Prokaryotic Cyanobacteria dominated the epilimnion communities (relative abundances ranging from 47.5–95.7% of total phototrophs). In addition, the identification of eukaryotic chloroplast sequences belonging to unclassified Cryptophyta and Stramenopiles (3.4–45.8% of total Cyanobacteria) suggests a mixed eukaryotic/prokaryotic nature of such blooms (Fig 6B). The prokaryotic Cyanobacteria showed a remarkably low species level diversity, a hallmark of blooming phenomena [86], with the majority of sequences (41–95.3% of the total Cyanobacteria community) belonging to the genus *Prochlorococcus*. Members of the *Prochlorococcus* are ubiquitous in marine habitats [87, 88], and their occurrence in freshwater habitats like Grand Lake has been sporadically reported [89].

B. Heterotrophic phycosphere (PA) and free living (FL) communities: These are aerobic heterotrophic lineages (Fig 6C–6E) that were present in low abundance in March but became highly enriched in June/September samples, conceivably due to the increased organic matter input to the ecosystem by primary producers. Collectively, this group showed a high level of diversity and included (lineages that contributed ≥ 5% to the total community in all datasets) members of the phyla Planctomycetes (Fig 6C), Verrucomicrobia (Fig 6D), and the class Beta-Proteobacteria (Fig 6E). The phylum Planctomycetes progressively increased in relative abundance in the epilimnion from June to September, regardless of the physical state of the sample (FL or PA) (Fig 6C). Members identified belonged to the order Phycisphaerales, the genus *Planctomyces*, and the uncultured clades CL500-15, and OM19, all of which are commonly encountered in freshwater ecosystems in association with bacterioplankton and macroalgae [90–93]. The Verrucomicrobia showed a decrease in relative abundances in PA and FL samples as the bloom progressed from June to September (Fig 6D). Members identified belonged to the unclassified clade LD19, the genera *Opitutus* and *Luteolibacter*, candidatus “*Xiphinematobacter”*, and unclassified lineages within Puniceicoccaceae, all of which are commonly encountered in freshwater ecosystems [84, 94–96]. Members of the Beta-Proteobacteria decreased in abundances from June to September in both PA and FL fractions, but were significantly higher in the FL fraction in September (Fig 6A) (Student t-test P-value = 0.0001). Members of *Polynucleobacter*, a ubiquitous and abundant lineage in freshwater habitats with representatives that exist both as free-living organisms as well as endosymbionts of freshwater ciliates [97], were significantly more abundant in the FL fraction in September (Student t-test P-value = 0.019), consistent with previous reports of its abundance in freshwater planktonic fraction [98]. On the other hand, members of the *Azohydromonas*, a heterotrophic polyhydroxybutrate-synthesizing bacterium commonly isolated from soil [99], as well as unclassified members of the family Comamonadaceae, common members of freshwater lakes communities [84], were more abundant in the PA fraction in September (Fig 6E).

C. Legacy microbial communities: These are lineages that represented a major component of March epilimnion communities and were still detected, albeit with much lower abundances, in June and September epilimnion samples (Fig 6F). These include Actinobacteria acl lineage and the *Flavobacterium* and unclassified Sphingobacteriales lineages within the Bacteroidetes. The magnitude of decrease in relative abundance was especially pronounced in PA communities.

#### 3.4.3. Thermocline and hypolimnion microbial community response to organic matter deposition and hypoxia/anoxia

Organic matter deposition and the development of hypoxic/anoxic conditions in the thermocline and hypolimnion greatly impacted the observed community structure patterns in deeper layers in Grand Lake in June and September. These samples were characterized by a marked increase in microbial diversity (Fig 3 and S2 Table) and gradual development of distinct PA and FL communities (Fig 4 and S3 Table), with such differences being more pronounced in September.

In general, microbial communities of PA and FL fractions in samples from deeper layers in June were fairly similar (Figs 4 and 7, average Bray Curtis dissimilarity indices = 0.42 ±0.1). The majority of samples of June hypolimnion microbial communities (76–90% of the total June benthic communities) were composed of lineages previously encountered in March samples, e.g. the phyla Actinobacteria, Bacteroidetes, as well as lineages encountered in June epilimnion samples (possibly through downward migration/deposition), e.g. Planctomycetes, Verrucomicrobia, and the beta and gamma classes of Proteobacteria (Fig 7A). In addition, distinct (previously unencountered) lineages were identified as a minor component of June thermocline and hypolimnion samples. These include candidatus “*Aquirestis”* within the Bacteroidetes, an aerobic slow-grower that commonly inhabits freshwater lakes [100], (Fig 7B), the genera *Crenothrix*, *Methylomonas*, within the Gamma-Proteobacteria (Fig 7C), and the genera *Methylotenera*, *Rhodoferax*, *Methyloversatilis*, and families Methyliphilaceae, Nitrosomonadaceae, and Rhodocyclaceae within the Beta-Proteobaceria (Fig 7D). The majority of these Proteobacteria lineages are commonly encountered in freshwater lakes and are essential for C1 compound metabolism, and methane cycling [101].

By September, anoxia is established in the lower layers that are also receiving an increased input of carbon (Fig 2). Therefore, analysis of thermocline and hypolimnion September samples, especially in the context of broader sampling schemes at various depths and locations as implemented in this study, provides a unique opportunity to identify PA and FL lineages that specifically developed to mediate organic carbon turnover under anoxic conditions in monomictic lakes. The microbial community in September exhibited the highest level of dissimilarity between thermocline and hypolimnion layers (average Bray Curtis dissimilarity indices = 0.8±0.07), as well as a significantly higher level of community dissimilarity between FL and PA communities within each layer (average Bray Curtis dissimilarity indices = 0.42±0.06) as opposed to June (average Bray Curtis dissimilarity indices = 0.34±0.06) and March (average Bray Curtis dissimilarity indices = 0.32±0.06) thermocline and hypolimnion samples (Student t-test p-value = 0.002) (Figs 3 and 4, S3 Table). In addition, the PA community in September hypolimnion samples was significantly more diverse than the FL community at the phylum level (Student t-test p-value = 0.0007). Within the PA thermocline and deep layer communities in September (Fig 8), 21±10.9% of the thermocline PA community and 9.3±1.5% of the hypolimnion PA community in September was made up of the legacy March and June components (Fig 8A). In addition, 13.7±8% of the thermocline PA community, and 4±0.6% of the hypolimnion PA community in September was made up of sinking primary producers encountered in epilimnion June samples (*Prochlorococcus* and Stramenopiles). More importantly, newer members of the community were identified that represent a native, authentic phycosphere community that is responding to this new input of materials. These include members of the Chloroflexi, “Latescibacteria” (previously candidate division WS3), Armatimonadetes, and Delta-Proteobacteria. Members of the phylum Chloroflexi (average abundance 1.5±0.6% in thermocline PA layers, and 6.3±2.7% in hypolimnion PA layers) mostly belonged to the genera *Caldilinea* and *Anaerolinea* (both chemoorganheterotrophs with variable oxygen tolerance and commonly isolated from hot springs [102, 103]), as well as the uncultured order H39 and class SOGA31. Members of the candidate phylum “Latescibacteria” showed an average abundance of 1.5±0.7% in thermocline PA layers and 2.7±2% in hypolimnion PA layers, with sequences affiliated with order Sediment-1 in the thermocline layers and the order GN03 in the hypolimnion layers. Analysis of representative genomic sequences of Candidate phylum “Latescibacteria” revealed possible connection to algal detritus degradation under anaerobic conditions [104]. Members of the Armatimonadetes (previously candidate phylum OP10) showed an average abundance of 0.14±0.11% in thermocline PA layers and 1±0.47% in hypolimnion PA layers. Armatimonadetes September PA communities were dominated by the families Armatimonadaceae in thermocline layers and Chthonomonadaceae in hypolimnion layers. Representatives of these families are chemoorganoheterotrophs that were previously isolated from freshwater [105], as well as soil [106] ecosystems. A highly diverse community of Delta-Proteobacteria was identified in September PA samples: Within the thermocline datasets, the majority of Delta-Proteobacteria sequences belonged to the order Myxococcales, fruiting gliding bacteria that thrive on water-insoluble organic matter, often dead or alive cells, when nutrients are plentiful [107], and the uncultured order MIZ46 previously found to be active during the generation of anoxia in freshwater systems [108], while in the deeper hypolimnion Delta-Proteobacteria sequences were dominated by sulfate reducers, presumably using sulfated compounds released from cell walls as electron acceptor (Desulfobacula and Desulfobacteraceae [109, 110]). The PA community of both thermocline and hypolimnion layers also contained significant levels of the predatory *Bdellovibrio*, a reflection of the increase in number of microbial preys [111]. In addition, the Fe-oxidizing Beta-Proteobacteria genus *Gallionella*, often encountered in freshwater ecosystems [112], the Verrucomicrobia lineages *Prosthecobacter* (a facultative anaerobe commonly isolated from both oligotrophic and eutrophic freshwater habitats [113]) and Verrucomicrobia subdivision 3 [114], the Bacteroidetes lineages *Cytophaga* (heterotrophic bacteria with preference for degrading biopolymers such as cellulose, chitin, and pectin) [80], Saprospiraceae (filamentous Bacteroidetes with heterotrophic mode of metabolism and cosmopolitan occurrence in freshwater lakes [115]), Holophagaceae (a strictly anaerobic heterotrophic family [116] harboring the genera *Holophaga* and *Geothrix*) were also identified.

Few notable exceptions were noted when comparing the phylum level (or class level in case of Proteobacteria) of the FL thermocline and hypolimnion community in September to the PA community discussed above. The FL community shows more of the legacy Actinobacteria, and less of the sedimenting Cyanobacteria. In addition, the FL thermocline and lower communities were enriched in several lineages compared to the PA, implicating their specific roles in degradation of metabolites secreted from the phycosphere-associated communities. These include the phylum Gemmatimonadetes, and the candidate phylum “Omnitrophica” (previously candidate phylum OP3), with average % abundance of 1.4±0.35%, and 1±0.3%, respectively (both of these lineages constituted < 1% in the thermocline and lower PA communities). The majority of the Gemmatimonadetes community (98.7±0.4%) was composed of the genus *Gemmatimonas*, previously suggested to contribute to the degradation and metabolism of high molecular weight organic matter following cyanobacterial bloom lysis [117], consistent with its abundance (>1%) in September samples in lower layers that are expected to harbor the lysed and sedimenting algal detritus. Similarly, the majority of “Omnitrophica” sequences (83.3±0.59%) belonged to the class PBS-25. Candidate phylum “Omnitrophica” is known to thrive in anoxic aquatic and terrestrial environments, consistent with its abundance in deep anoxic layers, and, based on single cell genomics, is thought to be heterotrophic [118, 119]. Other differences noted between the FL and PA deeper communities in September include significantly more *Methylomonas*, unclassified Phycisphaerales, *Opitutus*, Rickettsiales, Holophagaceae, MIZ46, and SOGA31, and significantly less *Planctomyces*, CL500-15, *Luteolibacter*, *Chondromyces*, and Chloracidobacteria in the FL fractions.

## 4. Discussion

In this study, we provide an overview of microbial community dynamics associated with seasonal blooming in a seasonally stratified lake. Our results highlight the dynamic nature and high level of spatiotemporal heterogeneity and complexity of the microbial community, identify the primary producers involved in the process and the associated epilimnion heterotrophic microbial community, as well as the microbial community mediating organic matter turnover under the newly developed hypoxic and anoxic conditions in the thermocline and hypolimnion. Sampling across several seasons, depths, and sites within the lake, as well as the separation of the particle-associated and the free-living fractions allowed us to describe a detailed inventory of the lake community structure and membership. Specifically, samples from several sites act as biological replicates and represent an accurate reflection of the gamma diversity in the lake, while sampling from various seasons and depths offer the opportunity of studying the effect of time and space on the microbial community especially in relation to the physical and chemical changes occurring in the lake. Finally, separation of the PA and FL fractions allowed for disentangling the effect of the phycosphere-associated community as well as the free-living community on the carbon turnover in the lake. The high level of diversity encountered in this study is in contrast to the relatively stable and low diversity communities described in studies with single-grab-sampling schemes [2].

In March, prior to lake stratification, the lake is oxygenated and completely mixed, and the microbial community identified is typical of freshwater lake ecosystems, e.g. Actinobacteria (acl clade), Bacteroidetes (*Flavobacterium*), and Verrucomicrobia [48, 96]. With very little primary production and low turbidity, no significant difference in the phylogenetic makeup was observed between the particle-associated and the free-living community. Collectively, microbial community analysis results from June and September samples argue for microbial succession. In June, the increasing temperatures stimulate primary producers (mainly *Prochlorococcus* and Stramenopiles), which in turn stimulate heterotrophic lineages in the epilimnion (members of the Planctomycetes, Verrucomicrobia, and Beta-Proteobacteria) either directly associated with the phycosphere, or free-living thriving on secreted organic matter or soluble metabolic low molecular weight products of the phycosphere. The process is associated with an increase in turbidity and chlorophyll-a levels. In September, the sedimentation of surface organic matter causes an increase in turbidity, and accelerates the development of hypoxia/anoxia in the thermocline and hypolimnion. This is reflected in the development of a distinct complex microbial community, with the enrichment of lineages previously rare in March and June samples, e.g. members of the Chloroflexi, Armatimonadetes, Candidatus “Latescibacteria”, and Delta-Proteobacteria in the PA fraction, and Gemmatimonadetes and Candidatus “Omnitrophica” in the FL fraction. The near absence of these lineages from other samples, along with the known metabolic capabilities of their members argue for their involvement in the degradation of organic matter under the newly developed anoxic conditions. The differences between PA and FL communities suggest that some lineages are more adapted to attachment to the sinking organic matter, e.g. “Latescibacteria” [104] and *Bdellovibrio* [120], while others are more likely to thrive on the products of the PA fraction metabolism. It is worth noting that while we only focused on the autocthonous carbon sources in this study (the phytoplankton), the contribution of allocthonous carbon input, e.g. effect of rain and flooding (Grand Lake experienced major flooding the year of sampling), should not be ignored and could have possibly contributed to the high turbidity values observed.

Our work identifies the major primary producers in Grand Lake. Interestingly, the major prokaryotic members belonged to the genus *Prochlorococcus*. This is fairly unexpected, since this genus, while known to be the smallest and most abundant photosynthetic organism with a near ubiquitous existence in marine habitats [87, 88], little to no occurrence of *Prochlorococcus* in freshwater ecosystems has been previously documented [89], especially a direct role in blooming, where prior studies identified *Microcystis* as the major cyanobacterial bloom member in freshwater lakes [121–123]. Factors influencing *Prochlorococcus* dominance and involvement in the blooming process in Grand Lake remain to be seen. Similarly, our work identifies members of the phyla Planctomycetes, Verrucomicrobia, and the class Beta-Proteobacteria as the major phycosphere-associated as well as free-living community developing in the epilimnion aerobic layer in response to blooming. The identity of the phycosphere microbial community has mostly been studied in the marine ecosystem [57, 65–69], and to a lesser extent in freshwater lakes [124–127]. The epilimnion communities in Grand Lake bear some similarity to freshwater bacterioplankton communities previously identified in four eutrophic lakes in Sweden [126], as well as communities in major rivers of the Mississippi River Basin [89]. However, due to the unusual occurrence of *Prochlorococcus* as the major cyanobacterial bloom member, Grand Lake epilimnion community was distinct from freshwater bacterioplankton communities dominated by *Microcystis* [124, 125, 127].

**Fig 9:**
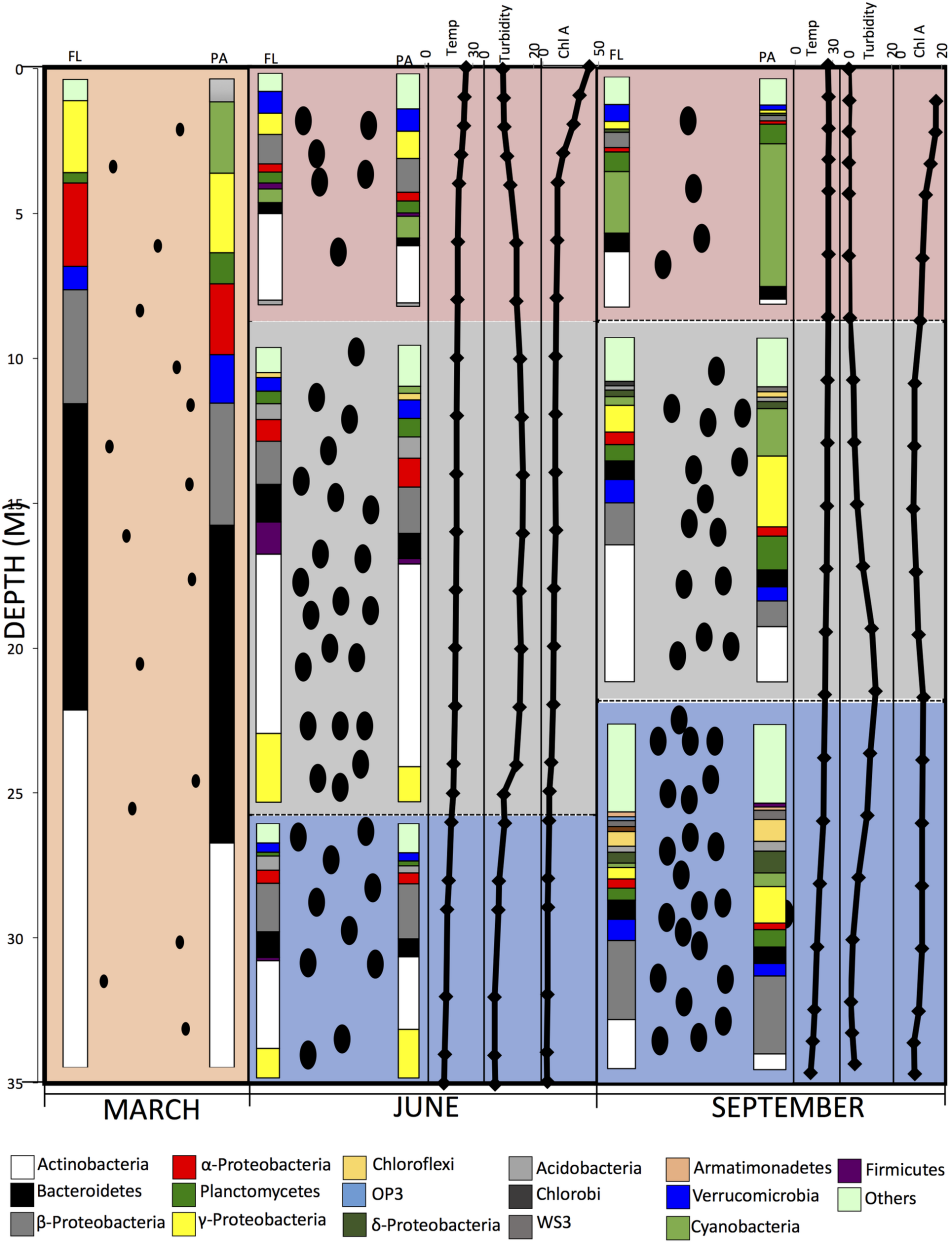
A model depicting the stratification process and the associated changes in dissolved O_2_ concentration in Grand Lake from a completely mixed oxic (pink) water column in March (left panel), to the stratification and the development of an oxic epilimnion (pink), a hypoxic thermocline (green), and a completely anoxic hypolimnion (blue) in June (thermocline panel) and September (right panel). The depth in (m) is shown to the left of the panels. The increase in the particulate concentration that happens with the algal blooming in June in the epilimnion along with the sedimentation of particles to the thermocline and the hypolimnion in June and September is shown as black ovals, the number of which in each layer (epilimnion, thermocline, and hypolimnion) is proportional to the turbidity and BGA cell counts. The size of the ovals is smaller and their number is lower in March to reflect the low turbidity and BGA counts pre-stratification. In June and September panels, the temperature (average temperature across the three sites studied, °C), turbidity (average turbidity across the three sites studied, NTU), and Chlorophyll A (average concentration across the three sites studied, μg/L) are shown (X-axis on top) versus depth (shown in M to the left of the panels). Also depicted is the average microbial community composition at the phylum level (or class level for Proteobacteria) for each of the sampling times and each of the sampling depths shown as % total abundance and grouped by sample physical state as FL or PA (shown on top). The phyla/class color-coding is shown at the hypolimnion. “Others” denote all phyla with < 1% total abundance, including the “unclassified” fraction (sequences that could not be classified with accuracy at the phylum level).

As described above, while the process of stratification and subsequent oxygen deprivation in deeper layers of seasonally stratified lakes is well described, surprisingly little is known about the microbial community associated with the process. Sampling the thermocline and hypolimnion in September represents an opportunity to identify the major microbial players, and elucidate lineages that have specifically developed in response to the process, especially by contrasting the benthic September microbial community to the epilimnion community in June, as well as to the legacy community in the lake. In addition, comparing the FL to PA benthic September communities, one could decipher which lineages were preferentially involved in direct versus indirect carbon turnover processes. The high diversity especially of the PA community highlights the involvement of multiple lineages in the process, and the differences between PA and FL communities argue for niche specialization. Our results (Figs 8 and 9) implicate that the chemoorganoheterotrophic facultative anaerobic or strictly anaerobic lineages within Bacteroidetes, “Latescibacteria”, Chloroflexi, and Actinobacteria are possibly directly involved in breaking down cell walls and high molecular weight polymer components within the attached phycosphere fraction, hence increasing its accessibility to other members of the community. Additional lineages, e.g. sulfate reducers, Myxobacteria, and Armatimonadetes would possibly take advantage of that and metabolize the exposed fraction within the attached phycosphere community, while the predatory *Bdellovibrio* would potentially cannibalize the released biomass. The low molecular weight organic matter and micronutrients released from the phycosphere will stimulate members identified in the FL fractions, e.g. Gemmatimonadetes, and “Omnitrophica”. Finally, methano- and methylotrophs in both the PA and the FL fractions, e.g. *Methylotenera* [128], *Methyloversatilis* [129], *Methylomonas* [130], and *Crenothrix* [131], and the families Methyliphilaceae [132], and Methylococcaceae [133], as well as members of the unclassified LD19 family of Verrucomicrobia (Fig 7E), would metabolize C1 compounds, and cycle methane [101]. We emphasize that the model is preliminary, and requires validation by possibly metagenomics/metatranscriptomics studies. The validity and applicability of this model to other ecosystems, and the impact of geochemistry, and the lake’s native community as well as allocthonous inputs remain to be seen in future studies.

## Supporting information

S1 Table Physical and chemical characteristics of Grand Lake. (DOCX)

S2 Table Diversity patterns. Diversity patterns were calculated for the datasets obtained both at the species level (0.03) and the order level (0.1). Datasets are grouped first by site, then by sampling time, then by sampling depth (Bot, hypolimnion; Mid, thermocline; and Surf, epilimnion), then by the sample physical state (PS: free-living, FL; particle-associated, PA).
(DOCX)

S3 Table Beta diversity indices. Bray Curtis dissimilarity indices for the beta diversity between datasets, where 0 denotes completely identical, while 1 denotes completely dissimilar communities. Numbers are averages obtained for the three sites studied.
(DOCX)
