# Supplementary material for: Spatiotemporal analysis of microbial community dynamics during seasonal stratification events in a freshwater lake (Grand Lake, OK, USA)
Source: PLoS One. 2017 May 11;12(5):e0177488. doi: 10.1371/journal.pone.0177488 (PMC5426677; doi:10.1371/journal.pone.0177488)
Supplement: S1 Table — (DOCX) [file pone.0177488.s001.docx]

**Table S1. Physical and chemical characteristics of Grand Lake.** Lake conditions as determined by the Grand River Dam Authority. ND = not determined.

| **Site** | **Month** | **Depth** | **Temp (°C)** | **DO (mg/L)** | **Cond (us/cm)** | **Turbidity (NTU)** | **TSS (mg/L)** | **BGA (cells/mL)** | **Pheo-a (µg/L)** | **Chl-a**  **(µg/L)** | **TN (mg/L)** | **TP (mg/L)** | **NH_3_-N (mg/L)** | **PO_4_ (mg/L)** | **NOX-N (mg/L)** | **Salinity (ppt)** | **pH** | **ORP (mV)** | **Hardness (mg/L)** | **Alkalinity (mg/L)** |
| --- | --- | --- | --- | --- | --- | --- | --- | --- | --- | --- | --- | --- | --- | --- | --- | --- | --- | --- | --- | --- |
| **Dream** | **March** | **Surf** | 7.55 | 13.93 | 279 | 0.9 | ND | 1412 | 123.00 | 4.5 | ND | ND | ND | ND | ND | 0.13 | 8.68 | 34.7 | ND | ND |
|  |  | **Mid** | 5.2 | 13.79 | 283 | 0.8 | ND | 1057 | ND | 7.2 | ND | ND | ND | ND | ND | 0.14 | 8.5 | 39.2 | ND | ND |
|  |  | **Bot** | 4.65 | 10 | 334 | 2.4 | ND | 1170 | ND | 3.1 | ND | ND | ND | ND | ND | 0.16 | 8.25 | 44.95 | ND | ND |
|  | **June** | **Surf** | 25.28 | 10.97 | 238 | 8.2 | 7.67 | 4056 | ND | 46.4 | 1.19 | 0.118 | 0.026 | 0.047 | 0.58 | 0.11 | 8.49 | 50.2 | 100 | 82.8 |
|  |  | **Mid** | 19.18 | 2.4 | 251 | 11.1 | ND | 595 | ND | 9.8 | ND | ND | ND | ND | ND | 0.12 | 6.98 | 77.5 | ND | ND |
|  |  | **Bot** | 11.97 | 0.21 | 335 | 4.7 | ND | 397 | ND | 4.1 | 1.19 | 0.092 | 0.059 | 0.059 | 0.983 | 0.16 | 7.13 | 73.8 | ND | ND |
|  | **Sep** | **Surf** | 26.89 | 7.56 | 276 | 0.3 | 3.1 | 8491 | ND | 20.6 | 0.513 | 0.048 | 0.003 | 0.028 | 0.047 | 0.13 | 8.76 | -27.5 | 131.2 | 102.4 |
|  |  | **Mid** | 24.15 | 0.18 | 274 | 12 | ND | 827 | ND | 9 | ND | ND | ND | ND | ND | 0.13 | 8.03 | -25.4 | ND | ND |
|  |  | **Bot** | 15.26 | -0.04 | 329 | 2 | ND | 738 | ND | 7.7 | 1.48 | 0.584 | 0.893 | 0.502 | 0.013 | 0.16 | 15.74 | -449.4 | 144.8 | 126.8 |
| **P. Dam** | **March** | **Surf** | 6.31 | 13.5 | 280 | -3.1 | ND | 692 | 71.00 | 3.5 | ND | ND | ND | ND | ND | 0.13 | 8.44 | 41.5 | ND | ND |
|  |  | **Mid** | 5.63 | 12.94 | 281 | 0.7 | ND | 777 | ND | 4.8 | ND | ND | ND | ND | ND | 0.13 | 8.35 | 43.1 | ND | ND |
|  |  | **Bot** | 4.96 | 12.03 | 282 | 4.3 | ND | 1027 | ND | 4 | ND | ND | ND | ND | ND | 0.13 | 8.15 | 47.8 | ND | ND |
|  | **June** | **Surf** | 24.74 | 8.46 | 239 | 8.7 | 7.47 | 2117 | ND | 31.4 | 1.18 | 0.122 | 0.019 | 0.05 | 0.647 | 0.11 | 7.85 | 79.6 | 97.6 | 78.8 |
|  |  | **Mid** | 19.69 | 2.79 | 241 | 12.6 | ND | 642 | ND | 10 | ND | ND | ND | ND | ND | 0.11 | 6.99 | 87.7 | ND | ND |
|  |  | **Bot** | 13.19 | 0.6 | 331 | 3.7 | ND | 170 | ND | 4.7 | 1.26 | 0.087 | 0.02 | 0.06 | 0.985 | 0.16 | 7.14 | 80.8 | ND | ND |
|  | **Sep** | **Surf** | 26.85 | 7.21 | 274 | -0.9 | 2.8 | 334 | ND | 0.3 | 0.500 | 0.044 | 0.004 | 0.027 | 0.055 | 0.13 | 8.7 | -17.2 | 128.4 | 104.4 |
|  |  | **Mid** | 24.03 | 0.12 | 261 | 8.8 | ND | 925 | ND | 11.1 | ND | ND | ND | ND | ND | 0.12 | 8.08 | -28.2 | ND | ND |
|  |  | **Bot** | 12.32 | -0.09 | 356 | 2.8 | ND | 515 | ND | 7.1 | 1.08 | 0.404 | 0.627 | 0.427 | 0.008 | 0.17 | 16.53 | -459.3 | 146.8 | 127.6 |
| **Tree** | **March** | **Surf** | 7.44 | 14.24 | 286 | 0.9 | ND | 1597 | 91.80 | 5.2 | ND | ND | ND | ND | ND | 0.14 | 8.82 | 27.5 | ND | ND |
|  |  | **Mid** | 4.65 | 13.12 | 310 | 1.9 | ND | 759 | ND | 4.3 | ND | ND | ND | ND | ND | 0.15 | 8.43 | 39.8 | ND | ND |
|  |  | **Bot** | 4.33 | 7.97 | 385 | 0.5 | ND | 1312 | ND | 2.2 | ND | ND | ND | ND | ND | 0.18 | 8.3 | 42.1 | ND | ND |
|  | **June** | **Surf** | 27.31 | 13.9 | 241 | 5.4 | 10.26 | 4164 | ND | 44.6 | 1.25 | 0.135 | 0.022 | 0.026 | 0.403 | 0.11 | 8.94 | 37.7 | 96.8 | 83.6 |
|  |  | **Mid** | 19.89 | 2.68 | 223 | 17.1 | ND | 775 | ND | 12.1 | 1.40 | 0.179 | 0.022 | 0.114 | 0.957 | 0.11 | 6.98 | 78.6 | ND | ND |
|  |  | **Bot** | 13.83 | 0.26 | 337 | 9.5 | ND | 375 | ND | 4.9 | 1.40 | 0.109 | 0.104 | 0.07 | 0.978 | 0.16 | 7.07 | 71 | ND | ND |
|  | **Sep** | **Surf** | 26.48 | 7.39 | 291 | -4.6 | 3.1 | 526 | ND | -0.4 | 0.623 | 0.07 | 0.007 | 0.047 | 0.151 | 0.14 | 9.32 | -58.5 | 127.2 | 114.0 |
|  |  | **Mid** | 25.48 | 2.36 | 309 | 5.4 | ND | 407 | ND | 5.5 | 0.954 | 0.112 | 0.013 | 0.089 | 0.549 | 0.15 | 8.21 | -30.6 | 133.6 | 109.6 |
|  |  | **Bot** | 21.03 | 0.06 | 274 | 8.5 | ND | 688 | ND | 9.8 | 1.46 | 0.419 | 0.825 | 0.35 | 0.054 | 0.13 | 10.65 | -261 | 121.6 | 111.2 |
