# Supplementary material for: Spatiotemporal analysis of microbial community dynamics during seasonal stratification events in a freshwater lake (Grand Lake, OK, USA)
Source: PLoS One. 2017 May 11;12(5):e0177488. doi: 10.1371/journal.pone.0177488 (PMC5426677; doi:10.1371/journal.pone.0177488)
Supplement: S2 Table — Diversity patterns were calculated for the datasets obtained both at the species level (0.03) and the order level (0.1). Datasets are grouped first by site, then by sampling time, then by sampling depth (Bot, hypolimnion; Mid, thermocline; and Surf, epilimnion), then by the sample physical state (PS: free-living, FL; particle-associated, PA). (DOCX) [file pone.0177488.s002.docx]

**Table S2.** Diversity patterns calculated for the datasets obtained both at the species level (0.03) and the order level (0.1). Datasets are grouped first by site, then by sampling time, then by sampling depth (Bot, hypolimnion; Mid, thermocline; and Surf, epilimnion), then by the sample physical state (PS: free-living, FL; particle-associated, PA).

|  |  |  |  | **# OTU's** | | **Coverage** | | **Shannon** | | **Ace** | | **Rarefaction Rank** | |
| --- | --- | --- | --- | --- | --- | --- | --- | --- | --- | --- | --- | --- | --- |
| **Site** | **Month** | **Depth** | **PS** | **0.03** | **0.1** | **0.03** | **0.1** | **0.03** | **0.1** | **0.03** | **0.1** | **0.03** | **0.1** |
| **Dream** | **March** | **Surf** | **FL** | 623 | 134 | 0.994 | 0.998 | 2.8 | 1.6 | 564 | 138 | 18 | 18 |
|  |  |  | **PA** | 676 | 169 | 0.989 | 0.997 | 2.8 | 1.5 | 1053 | 241 | 17 | 17 |
|  |  | **Mid** | **FL** | 1151 | 459 | 0.989 | 0.995 | 4 | 3.1 | 1185 | 536 | 16 | 16 |
|  |  |  | **PA** | 763 | 282 | 0.988 | 0.995 | 4.2 | 3.3 | 1377 | 588 | 14 | 15 |
|  |  | **Bot** | **FL** | 436 | 192 | 0.987 | 0.993 | 3.5 | 2.3 | 1600 | 720 | 15 | 14 |
|  |  |  | **PA** | 781 | 331 | 0.983 | 0.991 | 4.1 | 3.1 | 2002 | 951 | 13 | 13 |
|  | **June** | **Surf** | **FL** | 1442 | 544 | 0.980 | 0.990 | 4.6 | 3.6 | 1832 | 1152 | 11 | 11 |
|  |  |  | **PA** | 712 | 286 | 0.986 | 0.993 | 4.8 | 3.9 | 1345 | 734 | 12 | 12 |
|  |  | **Mid** | **FL** | 1322 | 508 | 0.978 | 0.990 | 3.7 | 3 | 1880 | 922 | 9 | 10 |
|  |  |  | **PA** | 2731 | 933 | 0.966 | 0.984 | 5 | 4.1 | 3683 | 1700 | 6 | 8 |
|  |  | **Bot** | **FL** | 1684 | 683 | 0.964 | 0.982 | 4.9 | 3.8 | 3431 | 1793 | 5 | 5 |
|  |  |  | **PA** | 1996 | 844 | 0.954 | 0.974 | 5.1 | 4 | 4655 | 2556 | 4 | 4 |
|  | **Sep** | **Surf** | **FL** | 1365 | 541 | 0.978 | 0.990 | 4.2 | 3.7 | 1880 | 844 | 10 | 9 |
|  |  |  | **PA** | 1696 | 687 | 0.974 | 0.986 | 4.4 | 3.8 | 2325 | 1250 | 8 | 7 |
|  |  | **Mid** | **FL** | 1804 | 789 | 0.971 | 0.984 | 4.7 | 3.8 | 2637 | 1614 | 7 | 6 |
|  |  |  | **PA** | 3329 | 1334 | 0.950 | 0.972 | 5.5 | 4.8 | 5302 | 3009 | 3 | 3 |
|  |  | **Bot** | **FL** | 3085 | 1867 | 0.917 | 0.954 | 6 | 5.1 | 7865 | 3919 | 1 | 1 |
|  |  |  | **PA** | 2749 | 1012 | 0.927 | 0.965 | 5.9 | 5 | 6495 | 2845 | 2 | 2 |
| **P. Dam** | **March** | **Surf** | **FL** | 770 | 291 | 0.990 | 0.995 | 3.6 | 2.4 | 1062 | 606 | 15 | 15 |
|  |  |  | **PA** | 764 | 322 | 0.987 | 0.994 | 4.2 | 3.3 | 1388 | 567 | 13 | 13 |
|  |  | **Mid** | **FL** | 512 | 174 | 0.992 | 0.997 | 2.3 | 1.6 | 865 | 288 | 16 | 16 |
|  |  | **Bot** | **PA** | 896 | 302 | 0.986 | 0.994 | 2.7 | 1.6 | 1424 | 527 | 14 | 14 |
|  | **June** | **Surf** | **FL** | 1447 | 529 | 0.981 | 0.992 | 4.5 | 3.5 | 1757 | 669 | 11 | 11 |
|  |  |  | **PA** | 55 | 38 | 0.239 | 0.507 | 4.6 | 3.7 | 371 | 248 | 12 | 12 |
|  |  | **Mid** | **FL** | 1230 | 425 | 0.970 | 0.987 | 4.9 | 3.8 | 2949 | 1285 | 5 | 9 |
|  |  |  | **PA** | 1839 | 657 | 0.974 | 0.988 | 4.9 | 3.9 | 2618 | 1378 | 7 | 8 |
|  |  | **Bot** | **FL** | 1437 | 587 | 0.975 | 0.987 | 4.6 | 3.6 | 2266 | 1185 | 9 | 10 |
|  |  |  | **PA** | 2955 | 1170 | 0.962 | 0.982 | 4.6 | 3.5 | 3889 | 1671 | 4 | 4 |
|  | **Sep** | **Surf** | **FL** | 1346 | 574 | 0.978 | 0.989 | 4.4 | 3.8 | 1924 | 981 | 10 | 7 |
|  |  |  | **PA** | 1954 | 758 | 0.972 | 0.986 | 4.6 | 3.9 | 2808 | 1423 | 8 | 6 |
|  |  | **Mid** | **FL** | 2023 | 927 | 0.964 | 0.981 | 5.2 | 4.4 | 2895 | 1504 | 3 | 3 |
|  |  |  | **PA** | 2339 | 919 | 0.969 | 0.983 | 4.2 | 3.6 | 2741 | 1433 | 6 | 5 |
|  |  | **Bot** | **FL** | 3217 | 1301 | 0.936 | 0.967 | 5.6 | 4.8 | 6490 | 2828 | 2 | 2 |
|  |  |  | **PA** | 2270 | 922 | 0.940 | 0.969 | 5.7 | 4.9 | 5364 | 2498 | 1 | 1 |
| **Tree** | **March** | **Surf** | **FL** | 485 | 190 | 0.991 | 0.996 | 3.4 | 2.3 | 1048 | 472 | 14 | 14 |
|  |  |  | **PA** | 266 | 119 | 0.973 | 0.987 | 4.2 | 3.3 | 587 | 215 | 13 | 13 |
|  |  | **Bot** | **FL** | 284 | 86 | 0.996 | 0.999 | 3 | 2.7 | 375 | 102 | 16 | 16 |
|  |  |  | **PA** | 369 | 108 | 0.993 | 0.998 | 3 | 2.7 | 888 | 171 | 15 | 15 |
|  | **June** | **Surf** | **FL** | 1271 | 449 | 0.982 | 0.992 | 4.5 | 3.8 | 1655 | 780 | 11 | 11 |
|  |  |  | **PA** | 243 | 116 | 0.904 | 0.953 | 4.4 | 3.9 | 628 | 227 | 12 | 12 |
|  |  | **Mid** | **FL** | 1312 | 505 | 0.977 | 0.988 | 4.7 | 3.7 | 2135 | 1466 | 9 | 9 |
|  |  |  | **PA** | 1980 | 731 | 0.969 | 0.984 | 4.9 | 3.9 | 2821 | 1523 | 7 | 8 |
|  |  | **Bot** | **FL** | 1540 | 558 | 0.957 | 0.982 | 4.8 | 3.7 | 4951 | 1896 | 5 | 5 |
|  |  |  | **PA** | 2948 | 1103 | 0.949 | 0.974 | 5.2 | 4.1 | 6404 | 3177 | 3 | 4 |
|  | **Sep** | **Surf** | **FL** | 1190 | 497 | 0.980 | 0.989 | 4 | 3.5 | 1654 | 896 | 10 | 10 |
|  |  |  | **PA** | 1725 | 691 | 0.973 | 0.986 | 4.2 | 3.7 | 2380 | 1250 | 8 | 7 |
|  |  | **Mid** | **FL** | 1528 | 631 | 0.970 | 0.984 | 4.5 | 3.7 | 2715 | 1573 | 6 | 6 |
|  |  |  | **PA** | 3677 | 1401 | 0.955 | 0.976 | 5.4 | 4.6 | 4161 | 2235 | 4 | 3 |
|  |  | **Bot** | **FL** | 2892 | 1212 | 0.928 | 0.960 | 5.7 | 4.5 | 7629 | 4086 | 1 | 1 |
|  |  |  | **PA** | 2782 | 1113 | 0.939 | 0.967 | 5.6 | 4.5 | 6232 | 2949 | 2 | 2 |
