# Supplementary material for: Spatiotemporal analysis of microbial community dynamics during seasonal stratification events in a freshwater lake (Grand Lake, OK, USA)
Source: PLoS One. 2017 May 11;12(5):e0177488. doi: 10.1371/journal.pone.0177488 (PMC5426677; doi:10.1371/journal.pone.0177488)
Supplement: S3 Table — Bray Curtis dissimilarity indices for the beta diversity between datasets, where 0 denotes completely identical, while 1 denotes completely dissimilar communities. Numbers are averages obtained for the three sites studied. (DOCX) [file pone.0177488.s003.docx]

Table S3. Bray Curtis dissimilarity indices for the beta diversity between datasets, where 0 denotes completely identical, while 1 denotes completely dissimilar communities. Numbers are averages obtained for the three sites studied.

| Bray Curtis dissimilarity index | | | Surface | | | | | | Middle | | | | | | Bottom | | | | | |
| --- | --- | --- | --- | --- | --- | --- | --- | --- | --- | --- | --- | --- | --- | --- | --- | --- | --- | --- | --- | --- |
|  |  |  | FL | | | PA | | | FL | | | PA | | | FL | | | PA | | |
|  |  |  | M | J | S | M | J | S | M | J | S | M | J | S | M | J | S | M | J | S |
| Epilimnion | FL | M | 0 | 0.78 | 0.87 | 0.44 | 0.97 | 0.97 | 0.74 | 0.66 | 0.85 | 0.80 | 0.69 | 0.92 | 0.90 | 0.76 | 0.87 | 0.88 | 0.73 | 0.95 |
|  |  | J | 0.78 | 0 | 0.64 | 0.82 | 0.87 | 0.83 | 0.74 | 0.65 | 0.63 | 0.61 | 0.47 | 0.74 | 0.73 | 0.70 | 0.79 | 0.80 | 0.70 | 0.89 |
|  |  | S | 0.87 | 0.64 | 0 | 0.92 | 0.86 | 0.58 | 0.90 | 0.77 | 0.50 | 0.82 | 0.76 | 0.65 | 0.89 | 0.87 | 0.85 | 0.93 | 0.84 | 0.91 |
|  | PA | M | 0.44 | 0.82 | 0.92 | 0 | 0.94 | 0.93 | 0.75 | 0.73 | 0.91 | 0.89 | 0.75 | 0.94 | 0.92 | 0.78 | 0.89 | 0.86 | 0.80 | 0.94 |
|  |  | J | 0.97 | 0.87 | 0.86 | 0.94 | 0 | 0.90 | 0.85 | 0.86 | 0.86 | 0.65 | 0.90 | 0.86 | 0.84 | 0.86 | 0.91 | 0.87 | 0.88 | 0.94 |
|  |  | S | 0.97 | 0.83 | 0.58 | 0.93 | 0.90 | 0 | 0.97 | 0.92 | 0.82 | 0.86 | 0.88 | 0.67 | 0.98 | 0.94 | 0.90 | 0.92 | 0.92 | 0.88 |
| Thermocline | FL | M | 0.74 | 0.74 | 0.90 | 0.75 | 0.85 | 0.97 | 0 | 0.73 | 0.87 | 0.40 | 0.69 | 0.91 | 0.50 | 0.71 | 0.86 | 0.44 | 0.77 | 0.95 |
|  |  | J | 0.66 | 0.65 | 0.77 | 0.73 | 0.86 | 0.92 | 0.73 | 0 | 0.68 | 0.74 | 0.39 | 0.78 | 0.82 | 0.61 | 0.78 | 0.84 | 0.57 | 0.89 |
|  |  | S | 0.85 | 0.63 | 0.50 | 0.91 | 0.86 | 0.82 | 0.87 | 0.68 | 0 | 0.78 | 0.67 | 0.45 | 0.85 | 0.74 | 0.76 | 0.90 | 0.73 | 0.85 |
|  | PA | M | 0.80 | 0.61 | 0.82 | 0.89 | 0.65 | 0.86 | 0.40 | 0.74 | 0.78 | 0 | 0.66 | 0.78 | 0.40 | 0.60 | 0.83 | 0.26 | 0.66 | 0.93 |
|  |  | J | 0.69 | 0.47 | 0.76 | 0.75 | 0.90 | 0.88 | 0.69 | 0.39 | 0.67 | 0.66 | 0 | 0.75 | 0.81 | 0.58 | 0.77 | 0.84 | 0.58 | 0.88 |
|  |  | S | 0.92 | 0.74 | 0.65 | 0.94 | 0.86 | 0.67 | 0.91 | 0.78 | 0.45 | 0.78 | 0.75 | 0 | 0.92 | 0.80 | 0.76 | 0.90 | 0.76 | 0.80 |
| Hypolimnion | FL | M | 0.90 | 0.73 | 0.89 | 0.92 | 0.84 | 0.98 | 0.50 | 0.82 | 0.85 | 0.40 | 0.81 | 0.92 | 0 | 0.63 | 0.91 | 0.32 | 0.82 | 0.96 |
|  |  | J | 0.70 | 0.70 | 0.85 | 0.79 | 0.88 | 0.94 | 0.71 | 0.52 | 0.73 | 0.60 | 0.54 | 0.79 | 0.77 | 0 | 0.70 | 0.79 | 0.39 | 0.83 |
|  |  | S | 0.87 | 0.79 | 0.85 | 0.89 | 0.91 | 0.90 | 0.86 | 0.78 | 0.76 | 0.83 | 0.77 | 0.76 | 0.91 | 0.67 | 0 | 0.92 | 0.68 | 0.42 |
|  | PA | M | 0.88 | 0.80 | 0.93 | 0.86 | 0.87 | 0.92 | 0.44 | 0.84 | 0.90 | 0.26 | 0.84 | 0.90 | 0.32 | 0.74 | 0.92 | 0 | 0.83 | 0.96 |
|  |  | J | 0.73 | 0.70 | 0.84 | 0.80 | 0.88 | 0.92 | 0.77 | 0.57 | 0.73 | 0.66 | 0.58 | 0.76 | 0.82 | 0.39 | 0.68 | 0.83 | 0 | 0.79 |
|  |  | S | 0.95 | 0.89 | 0.91 | 0.94 | 0.94 | 0.88 | 0.95 | 0.89 | 0.85 | 0.93 | 0.88 | 0.80 | 0.96 | 0.82 | 0.42 | 0.96 | 0.79 | 0 |
